# Diapause induces functional axonal regeneration after necrotic insult in *C*. *elegans*

**DOI:** 10.1371/journal.pgen.1007863

**Published:** 2019-01-14

**Authors:** Mauricio Caneo, Victoria Julian, Alexandra B. Byrne, Mark J. Alkema, Andrea Calixto

**Affiliations:** 1 Centro de Genómica y Bioinformática, Facultad de Ciencias, Universidad Mayor, Santiago de Chile, Chile; 2 Centro Interdisciplinario de Neurociencias de Valparaíso, Facultad de Ciencias, Universidad de Valparaíso, Valparaiso, Chile; 3 Neurobiology Department, University of Massachusetts Medical School, Worcester, MA, United States of America; HHMI, UC San Diego, UNITED STATES

## Abstract

Many neurons are unable to regenerate after damage. The ability to regenerate after an insult depends on life stage, neuronal subtype, intrinsic and extrinsic factors. *C*. *elegans* is a powerful model to test the genetic and environmental factors that affect axonal regeneration after damage, since its axons can regenerate after neuronal insult. Here we demonstrate that diapause promotes the complete morphological regeneration of truncated touch receptor neuron (TRN) axons expressing a neurotoxic MEC-4(d) DEG/ENaC channel. Truncated axons of different lengths were repaired during diapause and we observed potent axonal regrowth from somas alone. Complete morphological regeneration depends on DLK-1 but neuronal sprouting and outgrowth is DLK-1 independent. We show that TRN regeneration is fully functional since animals regain their ability to respond to mechanical stimulation. Thus, diapause induced regeneration provides a simple model of complete axonal regeneration which will greatly facilitate the study of environmental and genetic factors affecting the rate at which neurons die.

## Author summary

Diapause entry and hibernation have the striking ability to protect the nervous system from diverse types of damage. Here we show that the diapausing dauer larvae of *C*. *elegans* regenerate broken mechanosensory neurons that were damaged by the hyperactivation of degenerins (MEC-4d) or by axotomy during diapause. This regeneration is complete and functional, rendering neurons capable of responding to touch after three days in diapause. Genetic inactivation of the insulin receptor DAF-2 promotes regeneration of *mec-4d* axons in non-dauer animals. Overexpression of the downstream transcription factor DAF-16 promotes neuronal protection in *mec-4d* neurons while loss of *daf-16* accelerates *mec-4d* induced degeneration. Temperature sensitive activation of DAF-2 during diapause induces the loss of axonal integrity. This indicates that the insulin signaling pathway is an important underlying factor in regeneration. Additionally, we show that complete morphological regeneration depends on DLK-1, a conserved protein required for axonal repair. In this work we introduce a simple model of complete axonal regeneration, which will greatly facilitate the study of environmental and genetic factors affecting neurodegeneration and constitute an advantage in studying axonal regrowth.

## Introduction

Animals that enter diapause or hibernate not only survive for long periods of time without eating, but also preserve their nervous systems [1– 3]. This protection has been proposed to be a result of a high capacity for detoxification and maintenance of low oxidative stress [1– 4].

The bacterivore nematode *Caenorhabditis elegans* exits development and enters diapause under environmental stresses such as food deprivation, crowding, high temperatures, and exposure to pathogenic bacteria [5– 7]. Animals that enter diapause undergo a profound restructuring of physiology, morphology, metabolism and gene expression to accommodate lack of caloric intake. *C*. *elegans* animals that enter diapause become dauer larvae that live off fat reserves, which are tightly rationed by AMPK phosphorylation of lipase ATGL-1 [8]. In the absence of environmental stress, *C*. *elegans* undergo reproductive development and progress from embryos through four larval stages (L1–L4) to the adult stage. In contrast, dauers arrest progenitor cell lineages at a late-L2 like state which at the exit of quiescence, resume post dauer L3 fate [9]. Like L1-L2 larvae, dauers most likely use the glyoxylate cycle to generate gluconeogenic intermediaries from acetyl CoA [10].

We previously showed that in models of neuronal degeneration, neurons were completely protected in dauers. In this work, we use a simple model of axonal degeneration comprising a single neuron with a long process, a genetically encoded pro-degenerative stimulus and a simple behavioral test that assesses the function of a neuron, to study the environmental and genetic factors affecting axonal regrowth. Our model differs from axotomy in that the expression of hyperactive degenerins constitutes a chronic injury as opposed to the discrete damage produced by laser axotomy in multiple animals including *C*. *elegans* [11– 13], *Drosophila melanogaster* (fruit flies) [14, 15], *Danio rerio* (zebrafish) [16], and mammals [17]. We find that diapause strongly promotes the morphological and functional regeneration of mechanosensory axons that were broken by a genetically encoded trigger of chronic injury. Additionally, we show that wild-type dauers regenerate mechanosensory axons after a discrete injury conferred by laser axotomy. We further show that complete morphological and functional regeneration but not sprouting depends on the function of DLK-1.

## Results

### Diapause confers long term protection to damaged axons

**Fig 1:**
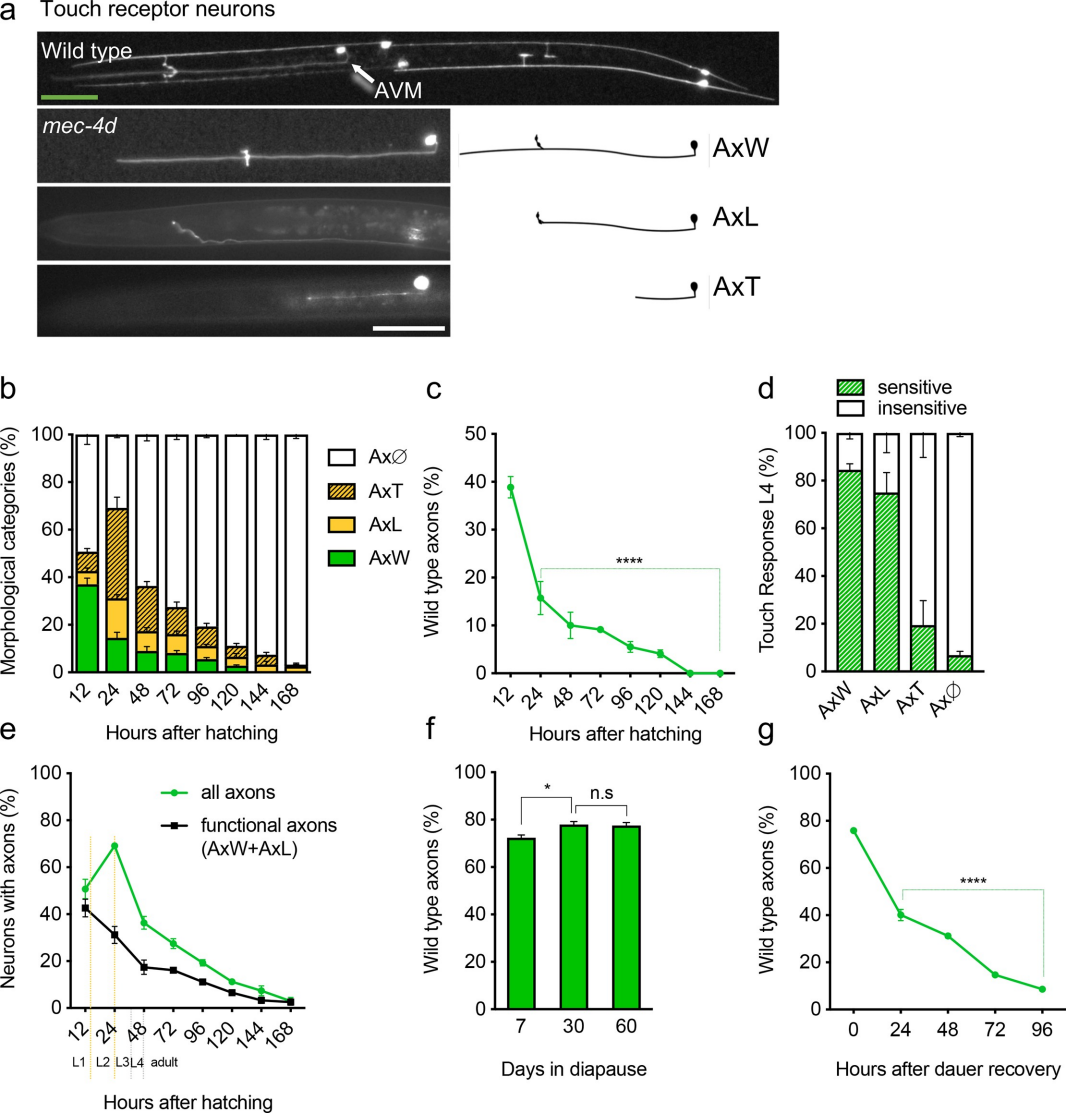
Diapause confers long term protection to damaged axons. A, *gfp* expressing touch receptor neurons in wild type and *mec-4d* animals with axonal categories of AVM neuron used in this Fig B, Time course progression of AVM degeneration in development of *mec-4d* mutants. C, Percent of animals with wild type AVM in B. D, Touch response of each morphological category in developing animals. E, Temporal course of functional categories and all axon in B. F-G, Percent of animals with wild type AVM at different times in diapause (F) and after dauer recovery (G). Scale bars represent 20 μm (orange bar for wild type panel, white bar for all *mec-4d* categories). P values ****< 0.0001, ***< 0.001, **<0.005, * <0.05. Error bars indicate the SEM in at least three biological replicas done in triplicates which had 30 or more animals each.

The expression of constitutively active degenerins such as *mec-4d(e1611)* and *deg-1(u38)* in *C*. *elegans* sensory neurons causes the necrotic death of those neurons [18– 21]. The *mec-4d* mutation, results in the expression of a dysfunctional MEC-4 pore-forming unit in the touch receptor neurons (TRNs) [22]. MEC-4d expression causes the unregulated entry of cations to the cell, provoking cell swelling, activation of proteases and the energetic collapse of the neurons [23, 24]. Embryonic TRNs, the Anterior Lateral Microtubule (ALM) and the Posterior Lateral Microtubule (PLM) neurons of the touch circuit arise between 400 to 500 minutes after the first zygote division in embryonic development [25]. In *mec-4d* mutants, embryonic TRNs have already died at the time of hatching, with somas of ALMs, PLMs and the Posterior Ventral Microtubule (PVM) vacuolated in early L1s [3, 24] (S1 Fig). The PVM neuron is also born at 12 hours after hatching in wild type animals. However, development of the PVM is difficult to analyze in the *mec-4d* background because only 3% and 4% of truncated axons are seen 48 and 72 hours after hatching, respectively, S1 Fig). The AVM (Anterior Ventral Microtubule) neuron is born 12 hours after hatching, and in *mec-4d* animals, degenerates in a time dependent manner and with a stereotyped change in morphology (Fig 1A and 1B, detailed description in Materials and Methods). First, the distal end of the axon breaks and small fragments of the severed axon degenerate (Axon Long, AxL). Subsequently, larger fragments of axon degenerate (Axonal Truncation, AxT), followed by the disappearance of all fragmented processes (Fig 1A and 1B and reference [3]).

To understand how AVM morphology correlates with function, we analyzed axon morphology of *mec-4d* L4 larvae using fluorescence microscopy and tested their ability to respond to touch [26]. The presence of wild-type and long AVM axons (AxW and AxL) was strongly correlated with function, while animals with truncated axons (AxT),without axons (either with (AxØ-S) or without a soma (AxØ) were largely touch insensitive (Fig 1D). While the number of functional axons (AxW and AxL) decreased with time, there was a brief increase in the number of animals with short axons (AxT) during the L2 stage (Fig 1E).

The time-dependent decline of wild-type axons observed in Fig 1C during development and adulthood is no longer observed if animals arrest as dauers in the late L2 larval stage, approximately 24–36 hours after hatching. After a week in the dauer stage, a large number of animals displayed wild-type axonal morphology that lasted for the duration of diapause (Fig 1F). Degeneration resumed immediately after dauer recovery (Fig 1G), suggesting that optimal conditions for neuroprotection and/or inhibition of degeneration that occur during diapause are lost as soon as animals feed *ad libitum*. Since the fraction of wild-type axons after 7 days in diapause (Fig 1F) was much higher than those present at 12 or 24 hours in normal development (Fig 1B and 1C), it is likely that during diapause, regrowth of axons may be the underlying reason for the observed protection.

### Diapause formation promotes the regeneration of damaged neurons

**Fig 2:**
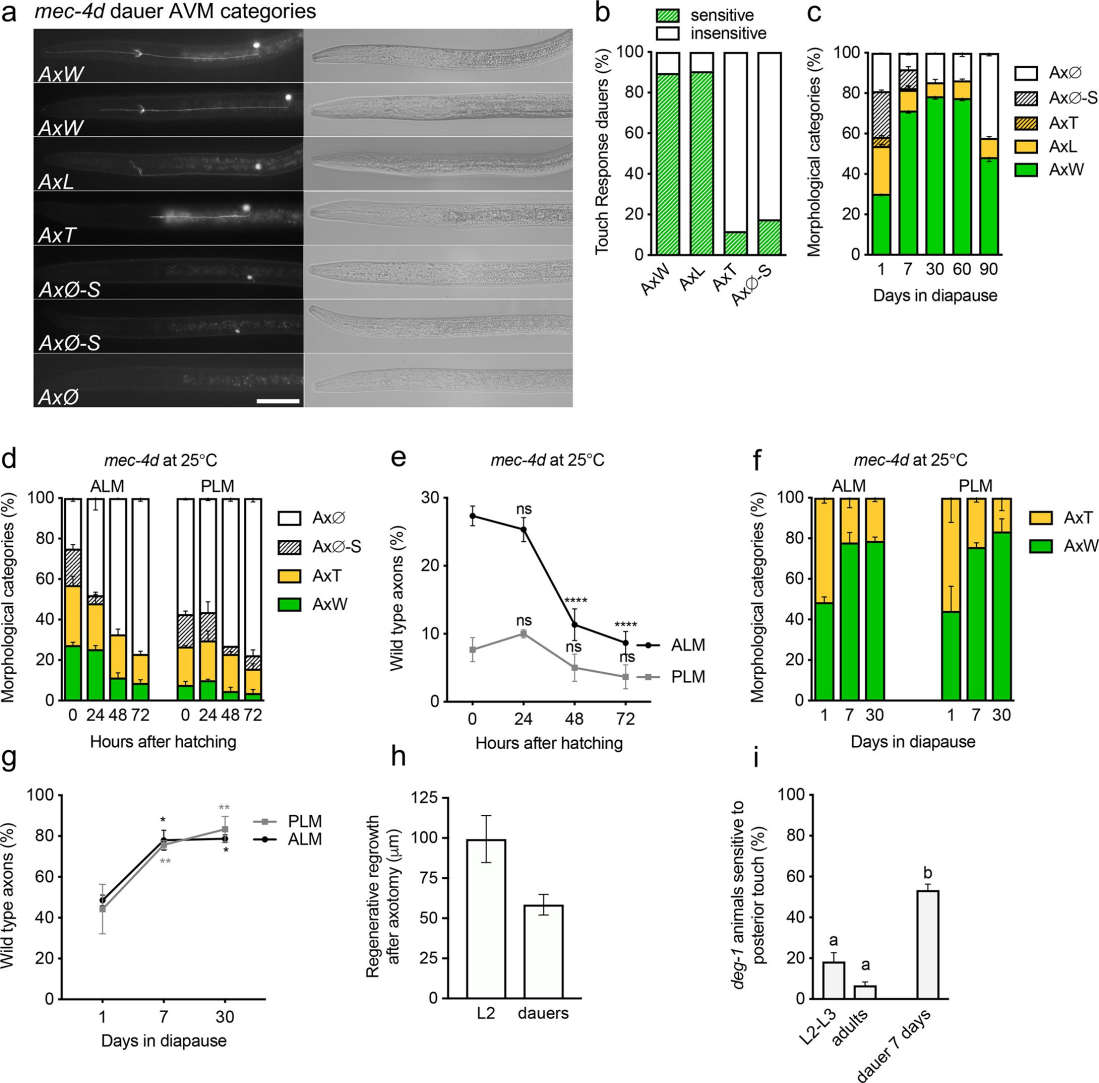
Diapause causes the regeneration of TRN axons. A, Morphological categories of the AVM neuron defined for dauer animals used throughout this work. B, Behavioral touch response of dauer animals with different morphological categories of axons. Significance of touch response (sensitive vs insensitive) in each axonal category was of P< 0.0001 and calculated using Chi square test. C, Time course of morphology of AVM in diapause after 7, 30, 60 and 90 days. D, Time course of embryonic *mec-4d* TRN degeneration at 25°C. E, Percent of animals with wild type embryonic TRN in D. F, Morphological categories of embryonic TRN neurons in *mec-4d* dauers after 1 day, 1 week and a month. G, Percent of animals with wild type embryonic TRN in F. H, Regrowth of wild type ALM neuron after 18–24 hours of laser axotomy, in dauers and L2 larvae. I, Posterior touch response of *deg-1(u38)* during development, adulthood and in diapause. Scale bars represent 20 μm. P values. ****< 0.0001, ***< 0.001, **<0.005, * <0.05. a and b denote that samples are significantly different, actual P values are given in Source S2 File. Error bars indicate the SEM in at least three biological replicas done in triplicates which had 30 or more animals each.

To test whether axons were able to regenerate during diapause, we assessed the morphological and functional changes of the *mec-4d* AVM neuron immediately after the formation of dauer larvae (first day of dauer, definition in Materials and Methods), and compared it to animals that had been in dauer for one week, 30, 60, or 90 days (Fig 2A, detailed description in Materials and Methods). To determine if the axonal morphology of the dauer *mec-4d* AVM correlated with function, we analyzed the animals’ ability to respond to touch to the anterior part of the body of dauer animals [3] and individually scored their AVM morphology. 90% of animals with wild-type axons (AxW) or axons with a process that extends to the nerve ring (AxL) were touch sensitive, while those with short axons (AxT) or without axons (AxØ-S or AxØ) were largely touch insensitive (Fig 2B). This shows that the ability to respond to gentle touch of a given morphological category is similar in dauers and developing larvae. The wild-type touch response of dauer *mec-4d* mutants suggests that MEC-4d channels are active during diapause [3].

The first day of dauer, 30.2% of animals had wild-type axons while on the seventh day of dauer this number increased to 71.4%, strongly suggesting that broken axons can regrow when animals are in diapause. The fraction of wild-type axons increased significantly after one month in diapause (to 78.5%, p = 0.002) and remained unchanged until 60 days in diapause (Fig 2C). The fraction of truncated neurons (AxL and AxT) decreased with time while the number of neurons with only a soma decreased at the 7^th^ day in diapause. At 90 days (3 months) in diapause, the number of wild-type axons decreased to 48.4%. This coincides with the limits of the lifespan of dauer animals. To test whether the decline of neuronal integrity in diapause was related to the expiration of dauer animals, we measured the lifespan of *mec-4d* dauer animals using N2 animals as controls. Only 10% of *mec-4d* dauer animals were still alive at day 90 (50% of wild type dauer animals were alive at day 90, S2 Fig). This may suggest that AVMs resume degeneration as a consequence of dauer animals reaching the end of their energy availability, which impacts on systemic and touch receptor neuron (TRN) ion homeostasis.

Can diapause entry stimulate regeneration of other axons? In *mec-4d* mutants the embryonic TRNs, ALM and PLM, died at the time of hatching [3, 24], and are therefore impractical for testing dauer regrowth. We previously observed that neurodegeneration of the TRNs was slower at 25°C compared to 20°C [3], providing an experimental setting to test the regrowth of embryonic neurons in diapause.

To examine the ability of embryonic touch cells to regenerate during diapause after *mec-4d* induced injury, we starved animals that had hatched at 25°C, a temperature at which approximately 50% of day 1 dauers had either truncated or wild type appearing ALM or PLMs (Fig 2F and 2G). We scored the neuronal integrity of dauer ALMs and PLMs at days 1, 7 and 30 of diapause. The observed increase in morphologically wild-type axons between the first and seventh day of diapause indicates that the ALMs and PLMs are capable of regenerating during diapause (Fig 2F and 2G). Specifically, 54% of animals had wild-type axons on the first day of diapause, while at 7 days this number significantly increased to 76%, and was maintained at 30 days. To confirm the ability of ALM axons to regenerate during diapause, we severed wild type ALM axons with a pulsed laser and measured regeneration 18–24 hours later. We found that like ALM axons chronically injured by *mec-4d* expression, ALM axons regenerated after being severed during diapause (Fig 2H).

Next, we tested whether diapause induced the functional regeneration of the PVC interneurons expressing the *deg-1* prodegenerative stimulus [18]. *deg-1* animals progressively lose the ability to respond to posterior touch due to the time dependent degeneration of the PVC interneuron [18]. We scored the ability of synchronized populations of *deg-1* animals (L2/L3 larvae, young adults and one-week dauers) to respond to posterior touch. While 18% and 7% of L2/L3 and adult animals, respectively, responded to tail touch, 53% of dauer animals responded to tail touch (Fig 2I), demonstrating functional regeneration of the PVC neuron. Our findings indicate that diapause strongly stimulates regeneration in two different sets of degenerating neurons.

### Fully regenerated neurons arise from cell somas

**Fig 3:**
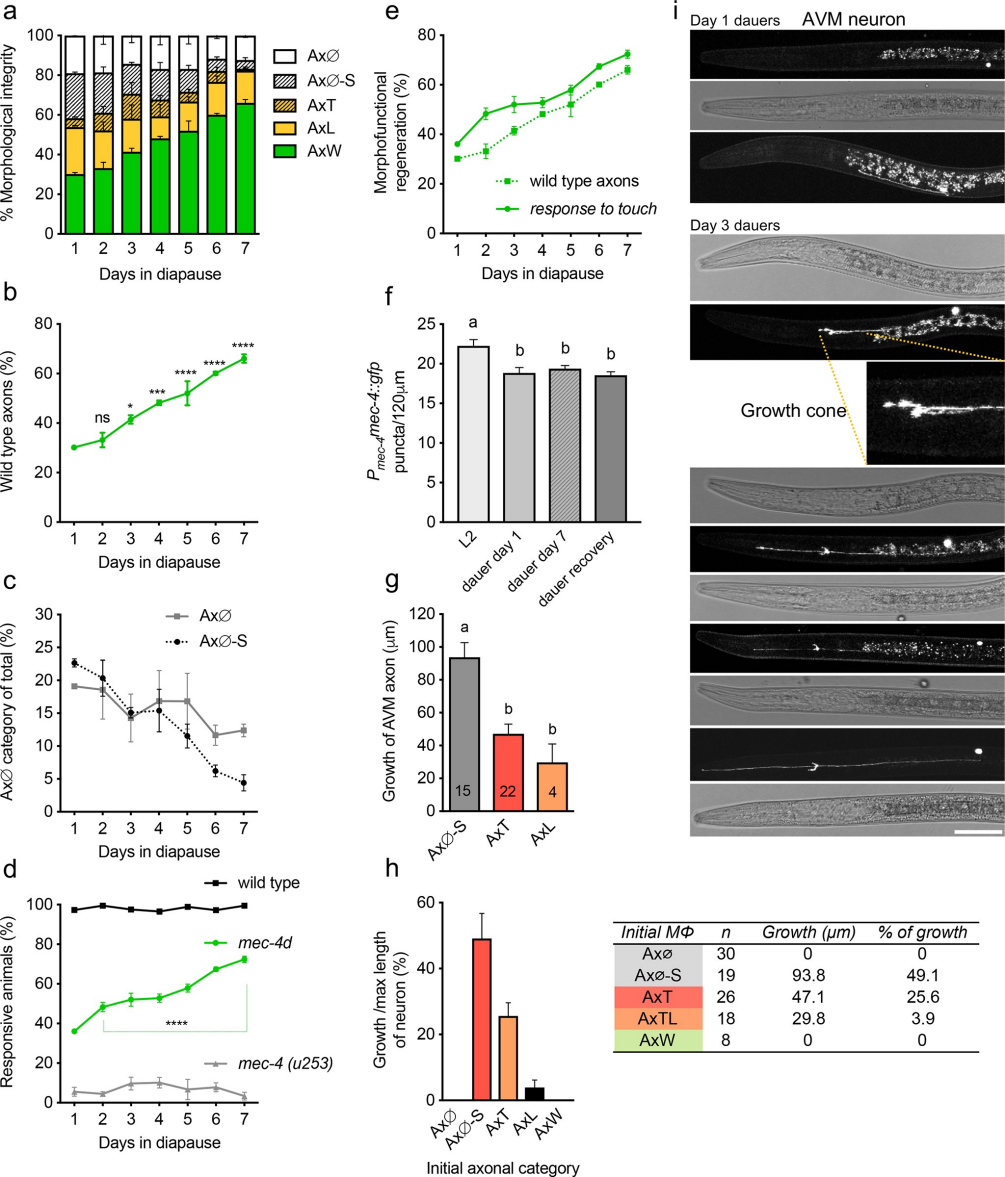
Functional and morphological regeneration of axons. A, Time course of morphology of AVM during the first week in diapause. B, Percent of wild type animals during the first week on diapause. Statistical analyses are comparisons with the first day in diapause. C, Temporal course of percent of neurons without axon, with (AxØ-S) or without a soma (AxØ). D, Percent of animals responsive to touch during the first week in diapause. E, Overlap between percent of wild type AVM neurons and touch response of the same *mec-4d* animals during the first week in dauer. F, Number of MEC-4::GFP puncta along the axon of the PLM neuron at 20°C in various life stages. G, Average growth of the three categories that displayed regrowth (see S1 Table); H, Percent of growth of each initial (first day of dauer) morphological category. The table below shows the number of animals counted on each category with a summary of the growth represented in B and C. I, High resolution confocal images depicting representative neurons at the indicated days during diapause of different animals. Scale bars represent 20 μm. P values ****< 0.0001, ***< 0.001, **<0.005, * <0.05. In panel F, a and b denote that samples are significantly different, actual P values are given in Source S2 File. Error bars indicate the SEM in at least three biological replicas done in triplicates. The N of experiments in G are included in the panel bars.

To further examine the AVM changes that occurred during the first seven days in diapause, we assessed AVM morphology and the ability to respond to gentle touch in populations of synchronized dauer animals every 24 hours (see Materials and Methods for details). The first significant change in the number of wild-type axons occurred on the third day of diapause compared to animals in the first day of diapause (41.4% vs 30.1%, respectively), reaching 60% on the seventh day of diapause (Fig 3A and 3B). The number of short truncated axons (AxT) decreased between days 1 and 4, and long axons (AxL) disappeared at day 7. Interestingly, the number of AVMs with only a soma (AxØ-S) decreased between day 1 and day 7, likely due to regrowth of their axon, while the number of animals with fully degenerated AVMs (no soma or axon present, AxØ), did not change in time (Fig 3A and 3C). This suggests that AVM neurons cannot arise *de novo* if their somas have degenerated, but that surviving AVM somas can regenerate an axon after *mec-4d* induced degeneration.

Importantly, the number of touch sensitive *mec-4d* animals increased during the first week of diapause (Fig 3D), which correlates with the increase in number of wild-type axons during the first week of diapause (Fig 3E). Wild-type (N2) dauer animals were touch sensitive showing that TRNs function normally during diapause, while *mec-4(u253)* loss-of-function mutants were completely insensitive. This demonstrates that the MEC-4 channel is required for mechanical response to gentle touch in diapause and it is therefore unlikely that other channels are contributing to the touch response in *mec-4d* dauer animals. We analyzed the distribution of MEC-4 channels along the TRN axons by measuring the number of MEC-4::GFP puncta in the PLM neuron. L2 animals exhibit an average of 22 puncta in 120 μm, while animals in dauer and at dauer exit had an average of 19 puncta per 120 μm (Fig 3F). This small yet significant difference does not diminish the degree of the touch response of animals (Fig 3D and 3F and S3 Fig).

To understand whether the initial morphology of axons impacts their ability to regenerate, we analyzed 71 individual dauers at the first and third day after diapause entry (details in Materials and Methods). The third day was the earliest time point when a significant increase in wild-type AVM morphology was observed in the population-based experiments (Fig 3B). When dauers resume development, they exit as L3/L4 larvae, which have larger pharynx than dauer animals. To ensure that animals remained at the dauer stage in our experiments we measured the pharyngeal width of *mec-4d* dauer animals, L3 animals and L4 animals (S4 Fig). The average pharyngeal width of dauer animals was 10.2 μm, while the average pharyngeal width of L3 and L4 animals was 20.6 and 24.3 μm respectively. Pharyngeal widths confirmed that animals followed longitudinally remained as dauers after three days. Average values for the width of the *mec-4d* animals’ pharynxes did not change between day 1 and day 3 of diapause and were approximately 11 μm (S2 Table), similar to the values for dauer animals (S4 Fig). On the third day, 65% of animals had regenerated their AVM axons (S1 Table), and 35% had not (S3 Table, pharyngeal measures in S4 Table). Importantly, the number of animals with wild-type axons examined longitudinally (40.8%) was similar to the 41.4% observed in the population-based studies (Fig 3B). Neurons with a soma but without an axon (AxØ-S) on the first day of diapause displayed the highest growth after three days, both in length (μm) and percentage of growth (Fig 3G and 3H). Fig 3I shows images of individual dauer animals with growing axons, starting at day 1 with only somas (AxØ-S) or truncated axons (AxT) and reaching wild-type length on the third day. Conversely, animals that entered diapause without neurons (30 individual AxØ animals) did not regrow a soma or axon (Fig 3H and S5 Table), which cannot be attributed to accidental exit from diapause (animals maintained a typical dauer morphology and narrow pharynx, S6 Table). Therefore, although dauer animals are capable of regenerating axons from a mechanosensory soma, they are not able to replace degenerated mechanosensory somas.

### ALM and PLM regrowth in dauer and development

Is the regenerative capacity of dauers comparable with that of developing animals, especially young larvae, which have a high capacity to regenerate [27]? Because *mec-4d* is a chronic prodegenerative stimulus and diapause entry is protective, the comparison between development and diapause required an acute stimulus, where degeneration was slowed or stopped after damage.

A stimulus that decreases degeneration is growing *mec-4d* animals at 25°C. At 25°C the ALM and PLMs did not degenerate during embryogenesis and are present after hatching and for several hours afterwards (Fig 2D and 2E). At 25°C, the AVMs also developed normally until 48–72 hours after hatching (S4 Fig and reference [3]). This allows the efficiency of regeneration of dauer animals to be compared to that of animals in development. Possible reasons for the protection of axons in *mec-4d* animals at 25°C include reduced MEC-4 channel expression, diminished function, and that a process required for degeneration downstream of channel activation is affected at higher temperatures.

**Fig 4:**
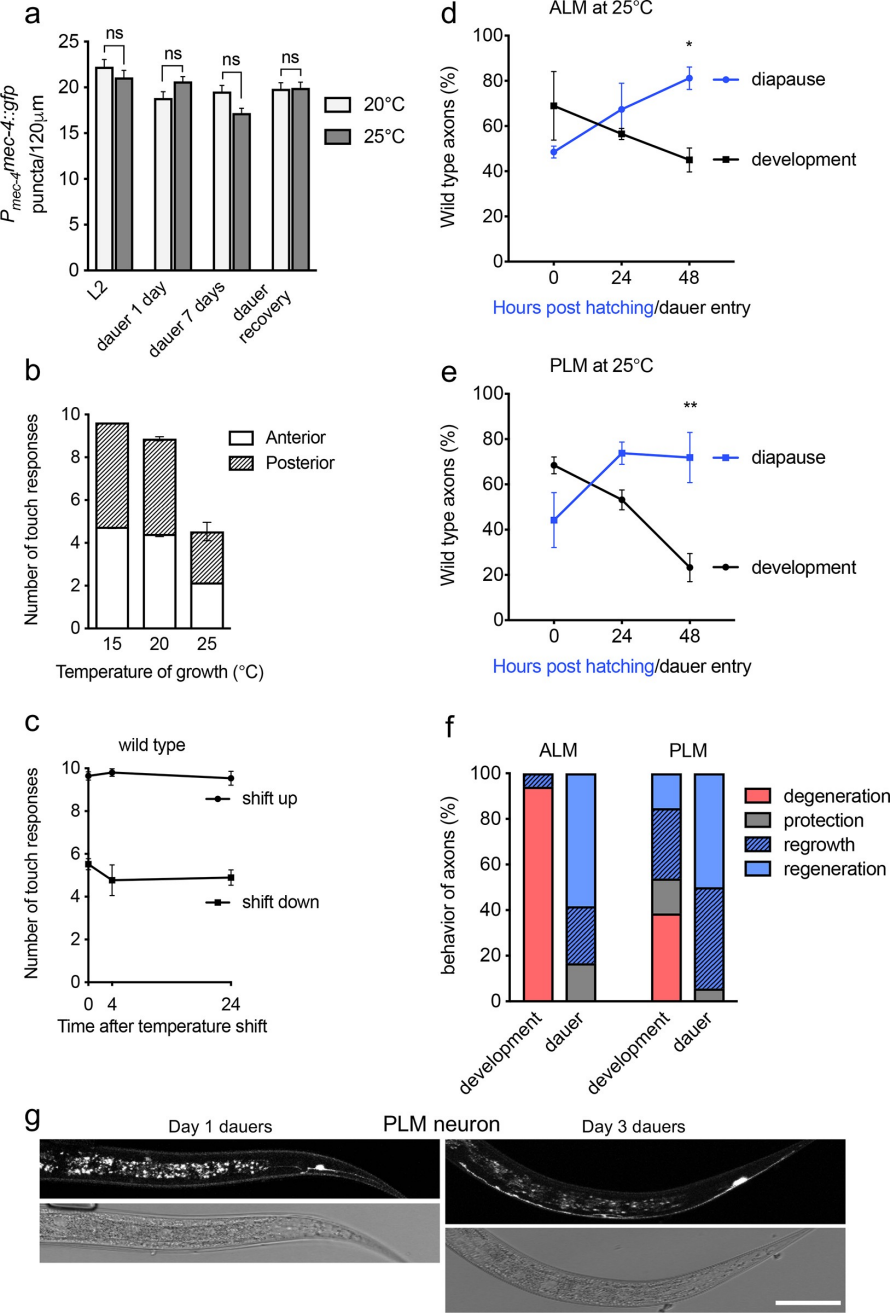
Individual assessment of regeneration in diapause. A, Comparison of the number of MEC-4::GFP puncta along the axon of the PLM neuron at 20 and 25°C in various life stages. B, Touch response of wild type animals at different temperatures of growth. C, Response to touch after temperature shift. Animals grown at 15°C were shifted to 25°C (shift up) and grown at 25°C and shifted to 15°C (shift down). D-E, Percent of animals with wild type ALM (D) and PLM (E) during diapause and development. F, Axonal behavior of ALM and PLM neurons during diapause and development. Axons that became shorter or disappeared were recorded as degeneration, those that remained the same size, as protection. Regrowth refers to growth without achieving wild type length, and regeneration was growth that resulted in restoration of wild type phenotype. G, High resolution confocal images depicting representative neurons at the indicated days during diapause. At day 1 dauer, a degenerated PLM neuron is shown. At day 3 dauer a regenerated PLM is shown. Scale bars represent 20 μm. P values ****< 0.0001, ***< 0.001, **<0.005, * <0.05. Error bars indicate the SEM in at least three biological replicas done in triplicates. N of panel F is of 30 individuals in dauer and 30 in development.

To test whether the expression of MEC-4 channels is affected at 25°C we measured the number of puncta in the PLMs of animals expressing MEC-4::GFP (see above and Materials and Methods) at 20ºC and 25ºC in L2, 1-day and 1-week dauer animals and at in animals that had exited the dauer stage(representative images of neurons at both temperatures are shown in S3 Fig). The number of MEC-4 channels in the TRN axons at each stage was similar at both temperatures, therefore expression of MEC-4 is not affected by temperature either in development or diapause (Fig 4A).

To assess mechanosensation at 25°C, we quantified the number of responses of developing animals to 10 anterior and posterior touches [26]. We found that wild-type animals responded to 100% of touches at 15°C and 20°C however animals responded 50% less frequently at 25°C (Fig 4B). This suggests animals raised at lower temperatures might habituate faster to mechanical stimulation than those raised at 25°C.

We next asked whether the decrease in the touch response observed at 25°C was due to transient or persistent molecular, cellular or biochemical change, which might include altered gene expression, protein localization, and protein modification. We grew animals at 15°C and 25°C until the L4 stage and performed touch tests immediately, 4 hours, and 24 hours after a temperature shift to 25°C or 15°C. After the temperature shift, animals responded with the same frequency that they had responded at the temperature they were raised (Fig 4C), suggesting that temperature induces a change that lasts for at least 24 hours. Taken together these results suggest that 25°C causes a long-lasting change that affects either the function of the MEC-4 channel itself or an intracellular process downstream of the MEC-4 channel.

To compare the growth of damaged axons during diapause with that of normal animal development, we chose the ALM and PLM touch receptor neurons. Unlike the AVM, the ALM and PLM complete development before hatching; therefore, axon regeneration can be evaluated independently of developmental axon outgrowth in the ALM and PLM. We compared the dynamics of *mec-4d*-dependent ALM and PLM axon degeneration in populations of worms (Fig 4D and 4E) and in individual worms (Fig 4F and 4G, and S7 and S8 Tables), during development and in diapause, at 25°C. We started with animals that had a soma with or without an axon (AxT, AxL, AxØ-S), excluding the AxØ category. In both the population and individual studies, the ALM and PLM axons degenerated during development, yet regrew in dauer animals (classification in Figure Legend), indicating diapause is a strong inductor of regeneration in the *mec-4d* degenerin model of degeneration.

### DAF-2 downregulation during diapause contributes to axonal maintenance

**Fig 5:**
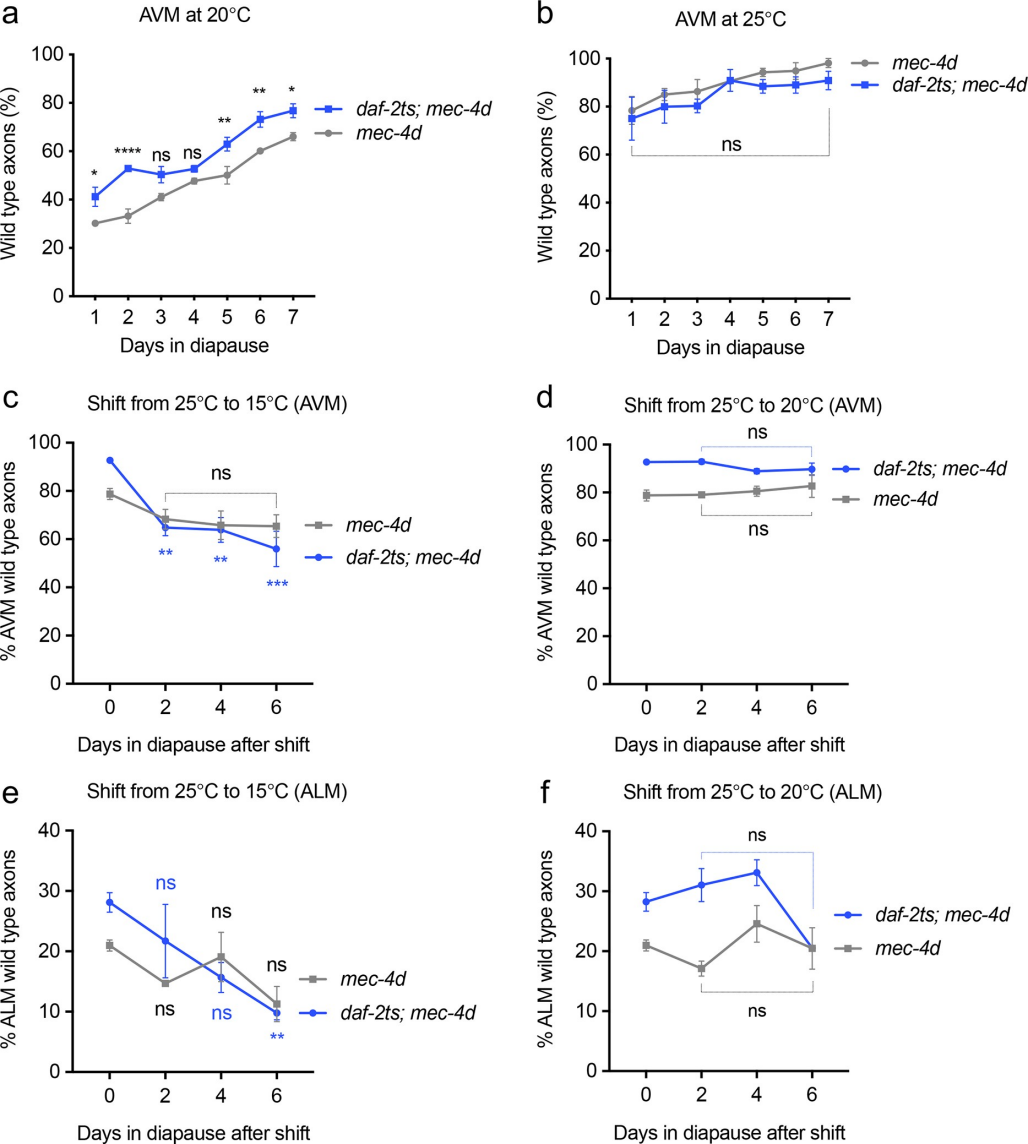
Effect of DAF-2 inactivation in dauer neuroprotection. A-B. Percent of *mec-4d* and *daf-2ts; mec-4d* animals with wild type AVM during the first week in diapause at 20°C (A) and 25°C (B). C-F Time course of *daf-2ts; mec-4d* AVM (C, D) and ALM (E, F) morphology after temperature shifts in diapause. Dauers formed at 25°C shifted to 15°C (C, E), or 20°C (D, F) scored at 2, 4 and 6 days after temperature shifts. P values ****< 0.0001, ***< 0.001, **<0.005, * <0.05. Error bars indicate the SEM in at least three biological replicas done in triplicates which had 30 or more animals each.

We next asked whether the molecular mechanisms that regulate dauer formation also regulate axon degeneration and repair in *mec-4d* axons. Dauer formation is accompanied by recognizable molecular changes such as the downregulation of the Insulin Receptor DAF-2 and the nuclear translocation of DAF-16/FOXO [28]. Not only does *daf-2* regulate dauer formation, it has also been shown to regulate axon regeneration after laser-induced injury [29– 31]. To determine whether loss of *daf-2* affects *mec-4d* induced changes in axon morphology in dauer animals, we performed a population-based time course analysis of axonal morphology of *daf-2ts; mec-4d* dauers every 24 hours for 1 week. The *daf-2ts* allele is a temperature sensitive mutation that reduces *daf-2* function at 25°C. Fig 5A shows that *daf-2ts; mec-4d* animals have significantly more wild-type axons at 20°C than *mec-4d* animals throughout the first week in diapause, except in days 3 and 4. A possible reason is that 20°C is semi-permissive for DAF-2 function and even though there are more wild-type axons in *daf-2; mec-4d*, they are not statistically different from *mec-4d*. At 25°C where DAF-2 is completely nonfunctional, both strains behaved similarly (Fig 5B). This suggests that at 25°C DAF-2 disfunction provided by dauer entry and the non-permissive temperature are inseparable. This, together with the hypofunction of MEC-4d (Fig 4B) render a highly protective environment. In an effort to further separate the global state of diapause from DAF-2 downregulation, we evaluated the neuronal integrity of AVMs and ALMs in dauers raised at 25°C and temperature shifted to 15°C or 20°C. Temperature shift of *daf-2ts; mec-4d* dauers but not *mec-4d* from the non-permissive (25°C) to the permissive (15°C) temperature significantly reduced the number of AVM and ALM wild-type axons during diapause (Fig 5C and 5E). Temperature shifts to 20°C, a semi-permissive temperature for insulin signaling, did not diminish dauer protection (Fig 5D and 5F). Importantly, shifting the temperature from 25°C to 15°C during diapause did not cause animals to exit the dauer state. Together, these results show that inactivation of DAF-2 regulates axonal maintenance in dauer animals.

### DAF-2 downregulation favors regeneration in non-dauer animals

**Fig 6:**
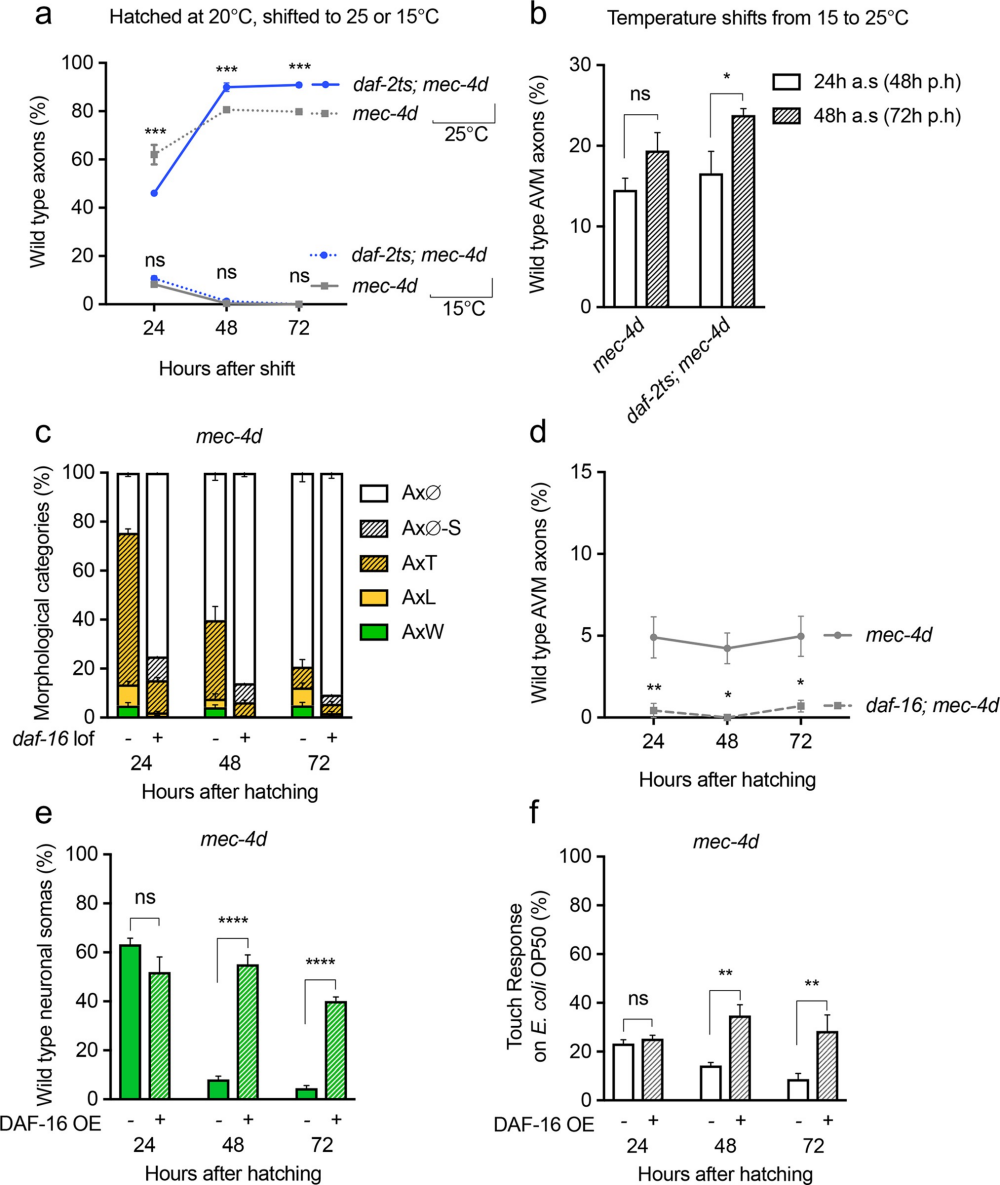
Effect of mutations in the insulin pathway on regeneration and protection in non-dauer animals. A- B Percent of *mec-4d* and *daf-2ts; mec-4d* animals with wild type AVM at 24, 48 and 72 hours after the indicated temperature shift at hatching (A); and at 24 or 48 hours after a temperature shift to 25°C. 24 and 48 hours after shift (a.s) correspond to 48 and 72 hours post hatching (p.s) (B). C-D, Time course of *daf-16; mec-4d* AVM morphology (C) and wild type axons (D). E- F Time course of degeneration (E) and touch response (F) of *mec-4d* animals expressing the *zIs356* transgene (DAF-16::GFP). P values ****< 0.0001, ***< 0.001, **<0.005, * <0.05. Error bars indicate the SEM in at least three biological replicas done in triplicates which had 30 or more animals each.

To test whether DAF-2 hypoactivity induced regrowth of the touch receptor neurons independently of dauer formation, *daf-2ts; mec-4d* animals hatched at 20°C were shifted to 15°C or 25°C as L1 larvae. To control for the protective effect of higher temperatures on the *mec-4d* induced degeneration (reference [3] and S5 Fig) we used *mec-4d* animals for comparison. *daf-2ts; mec-4d* animals shifted at birth to 25°C started with less wild-type axons at 24 hours than *mec-4d* mutants, but had a significantly larger number of AxW axons at 48 and 72 hours (Fig 6A). Axons in *daf-2ts; mec-4d* animals shifted to 15°C, where DAF-2 is fully functional, were indistinguishable from those in *mec-4d* mutants. These results suggest that DAF-2 downregulation contributes to neuronal protection [3] and potentially to axonal regrowth of *mec-4d* damaged axons.

To directly test whether DAF-2 downregulation promoted regrowth of axons, we performed temperature shifts at later stages of development when AVM was already born and had various degrees of degeneration. *daf-2ts; mec-4d* and *mec-4d* animals born at 20°C were kept for 24 hours at the permissive temperature of 15°C. After 24 hours at 15°C, animals were shifted to 25°C and their morphology was scored 24 and 48 hours later (Fig 6B). While in *mec-4d* animals the change in wild-type axons was not significantly different, *daf-2ts; mec-4d* animals regrew their AVM neurons 48 hours after the shift from 15°C to 25°C (72 hours after hatching, Fig 6B). This result suggests that DAF-2 downregulation promotes regrowth of axons in non-dauer *mec-4d* animals.

DAF-16 activation is a key downstream factor in the inhibition of regeneration caused by DAF-2 [29]. To test the role of *daf-16* in the degeneration of the TRN we compared the time course of degeneration in *mec-4d* animals to *daf-16; mec-4d* mutants. *daf-16* mutation caused a rapid degeneration of the *mec-4d* AVM, starting from 12 hours post hatching (Fig 6C and 6D). Conversely, transgenic overexpression of DAF-16 conferred significant protection to the integrity of the AVM (Fig 6E) and its function (Fig 6F). These results show that DAF-16 plays a crucial role in the protection of *mec-4d* axons conferred by DAF-2 downregulation.

### Loss of DLK-1 affects the degeneration dynamics of *mec-4d*-expressing neurons during development

**Fig 7:**
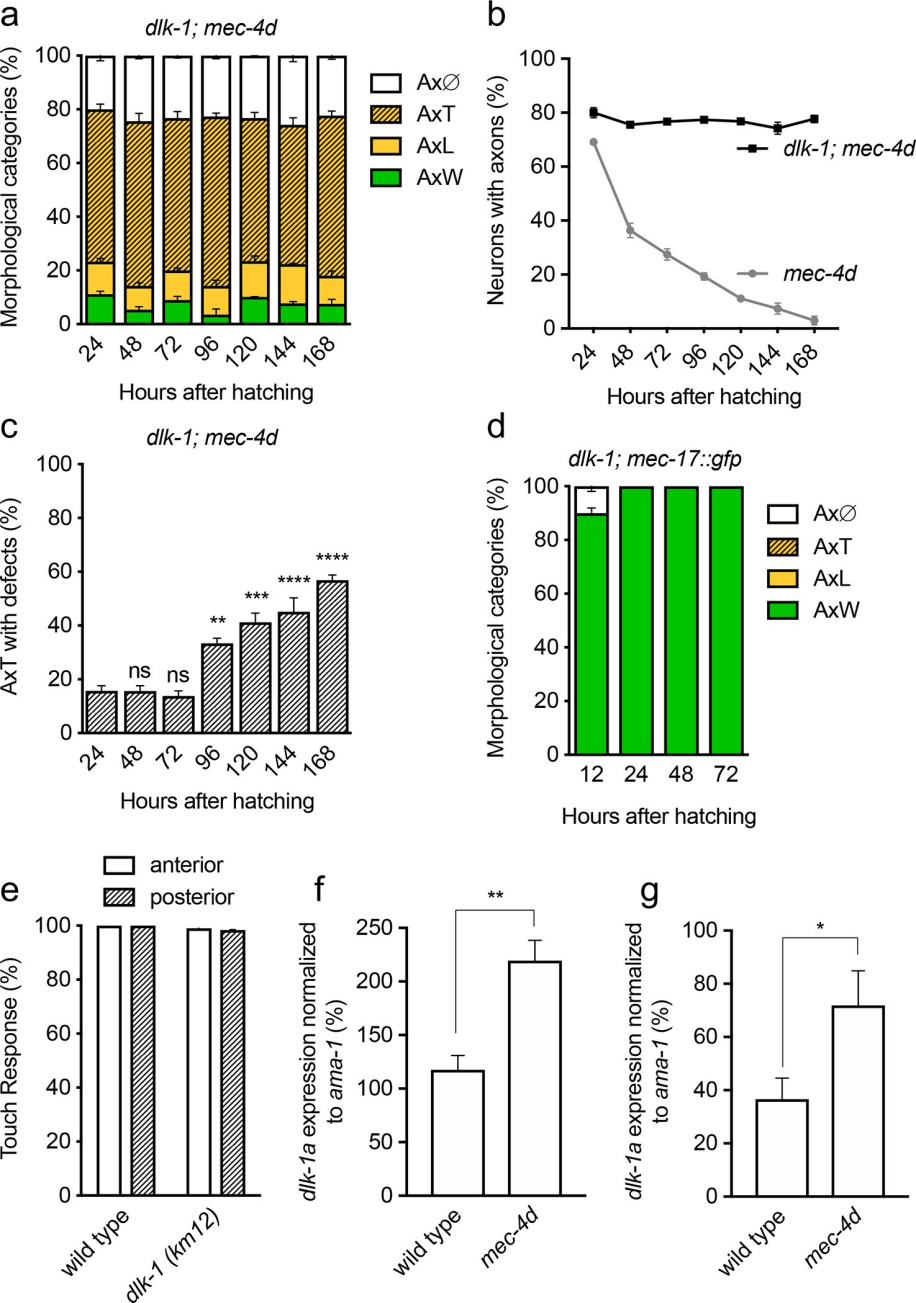
DLK-1 is required for degenerating *mec-4d* neurons. A, Time course of AVM *dlk-1; mec-4d* degeneration. B, Comparison of the total number of axons in *mec-4d* with the *dlk-1; mec-4d* double mutant during normal development and into adulthood. C, Temporal course of appearance of neurite defects in truncated *dlk-1; mec-4d* AVM neurons. D, Temporal course of AVM integrity in *dlk-1* mutants. E, Touch response of wild type animals and *dlk-1* mutants. (F-G) Percent of *dlk-1a* expression (normalized to *ama-1*) in wild type and *mec-4d* animals in development (F) and dauers (G). P values ****< 0.0001, ***< 0.001, **<0.005, * <0.05. Error bars indicate the SEM in at least three biological replicas done in triplicates which had 30 or more animals each.

The mitogen-activated protein kinase kinase kinase DLK-1 is essential for axon regeneration in models of axonal damage in *C*. *elegans* [13, 32], *Drosophila* [33], and mice [34–36] and has been implicated in neuronal development and degeneration in diverse organisms [37– 40]. We asked whether *mec-4d*-induced TRN degeneration and later diapause-induced regeneration in *C*. *elegans* require DLK-1 function. To answer the first question, we scored AVM morphology during normal development and in diapause in *dlk-1*(*km12*) loss-of-function mutants, in a wild-type or *mec-4d* background. During normal development of *mec-4d; dlk-1* animals, only 11.1% of the animals had wild-type AVM neurons at 24 hours, similar to the number of wild-type AVM neurons to *mec-4d* animal at 24 hours. However, unlike *mec-4d* animals, the number of wild-type axons in *mec-4d; dlk-1* animals did not change in adulthood and for several days after fertility (Fig 7A). This suggests that DLK-1 is needed for degeneration of the TRNs as shown for *Drosophila* and mice [37]. Fig 7B shows the number of AVM neurons remaining (the sum of all neurons with axons, regardless of category) for *mec-4d* and *dlk-1; mec-4d* animals during the course of development and adulthood. While *mec-4d* animals completely degenerated their axons, *dlk-1* loss impaired the normal course of degeneration. Most truncated (AxT) neurons in *dlk-1; mec-4d* mutants displayed neurite defects during development that increased as animals aged. Fig 7C shows the number of animals with defective AVM (neurons with posterior processes, double axons and random sprouting from cell soma and axon) within the AxT category in a temporal course. These defects were only visible in *dlk-1; mec-4d* animals, because *dlk-1* mutants did not display abnormalities in AVM development (*dlk-1; P*_*mec-17*_*mec-17gfp* in Fig 7D) or in touch response (Fig 7E). We also observed that *dlk-1* mRNA expression in *mec-4d* mutants is higher than in wild-type animals both in development (Fig 7F) and diapause (Fig 7G). Together, this shows that DLK-1 plays an important role in TRNs upon neuronal damage.

### Significant regeneration during diapause requires DLK-1

**Fig 8:**
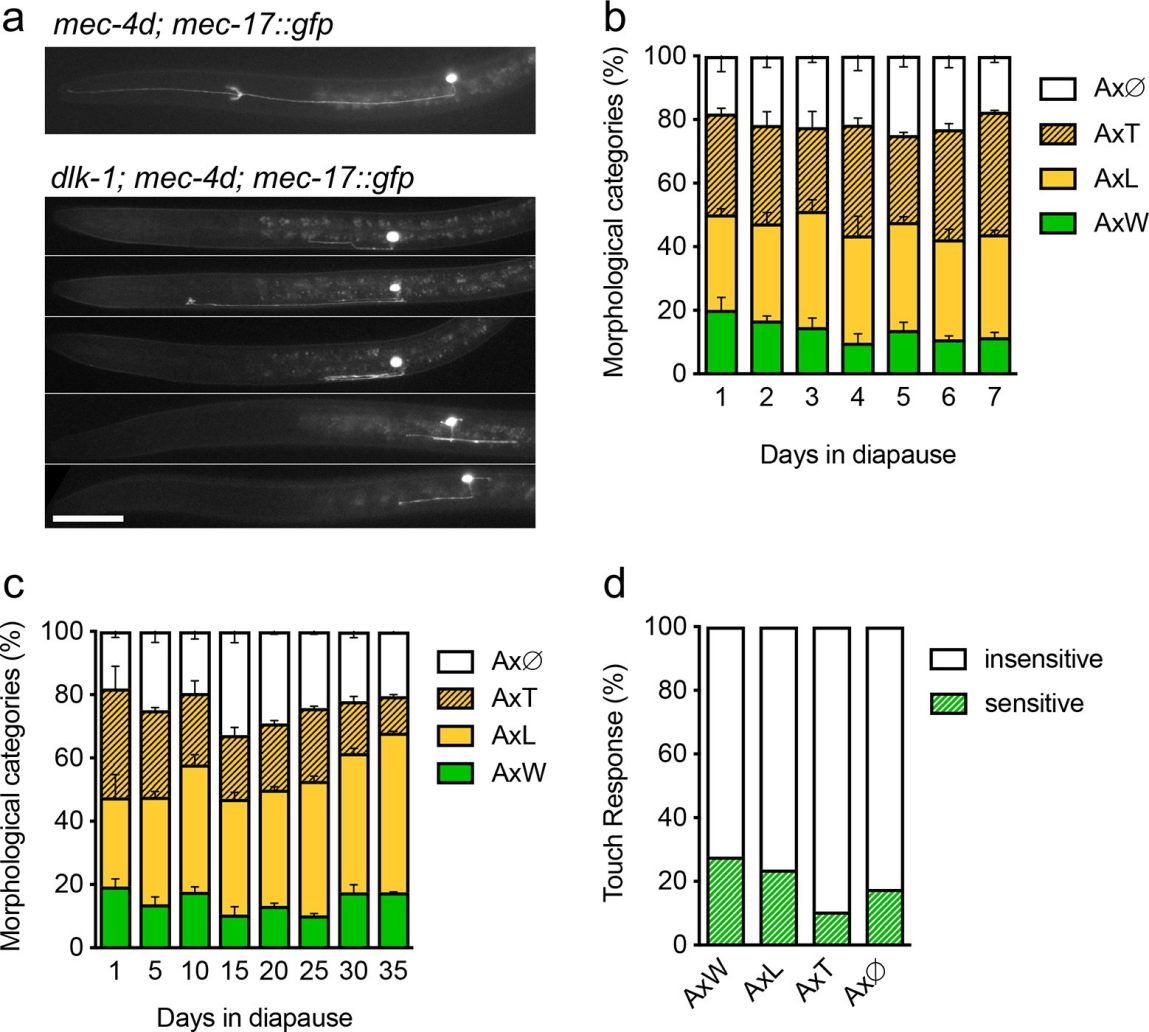
DLK-1 is required for functional regeneration of *mec-4d* neurons. A, Photographs of neurite defects observed in *dlk-1; mec-4d* dauers axons. B-C, Time course of AVM *dlk-1; mec-4d* dauers for one week (B) and longer times (C). D, Behavioral touch response of animals with different morphological categories of axons. Scale bars represent 20 μm.

To understand the role of DLK-1 in axon regeneration observed in diapause animals, we performed a population-based time course analysis of axonal growth in *dlk-1; mec-4d* dauer animals. Like non-dauer *dlk-1; mec-4d* animals a number of dauer animals also displayed neurite defects (Fig 8A). The number of wild-type axons (AxW) did not vary significantly between the first and seventh day, indicating that DLK-1 is needed for growth of axons in diapause (Fig 8B). Analysis of longer times into diapause showed that axonal categories remain mostly unchanged in *dlk-1; mec-4d* animals. Especially wild-type axons were not significantly different throughout the 35 days (Fig 8C). Given that loss of *dlk-1* rendered axons largely static; we wondered whether neurons that were technically capable of responding (AxL and AxW) did so. Touch tests were performed on *dlk-1; mec-4d* dauers that were subsequently mounted on agar pads for observation. AxW and AxL AVM neurons were non-functional in *dlk-1; mec-4d* mutants (Fig 8D), suggesting that even when their morphology is intact, the function of these neurons is impaired.

To further study the AVM dynamics of *dlk-1; mec-4d* animals, we analyzed and measured individual axons in a 3-day time course. S6 Table shows that animals stayed in diapause for the length of the experiment, as measured by the width of the pharynx. Even though *dlk-1; mec-4d* dauers were not capable of fully regenerating, some neurons extended small processes (S9 Table). The growth observed (18.2%) was primarily from axons that started as Ax∅ or AxT the first day of dauer and reached AxT or AxL. Importantly, 50% of the growth was erratic, with additional axonal sprouts from the cell body, double processes and neurites oriented posteriorly (Fig 8A and S9 Table). This shows that there is a small DLK-1 independent outgrowth of neurites in *mec-4d* axons, but full-length regeneration requires DLK-1 during diapause.

## Discussion

### Diapause fully repairs damaged axons

Dormancy in the form of diapause and hibernation allows the preservation of tissues and organs for long periods of time in complete absence of food intake. A common feature of different types of dormancy is the decrease in metabolic rate and gene expression [41– 45]. The nervous system is a remarkable example of tissue preservation during restoration of blood flow in hibernation [1, 2] and protection from injury during diapause [3]. In this work, we show that *C*. *elegans* undergoing diapause are capable of fully regenerating damaged touch receptor neurons expressing a constitutively active degenerin, MEC-4d. Neuronal growth in *mec-4d*-expressing animals is in most cases complete as 36% more axons have a wild-type morphology by the seventh day of diapause compared to the first day of diapause and 72% of axons are functional on the seventh day of diapause (Fig 3D). This protection is long lasting, as 86% of AVM neurons are functional after 2 months in diapause (Fig 2C). Strikingly, elongation of *mec-4d* AVM axons during diapause is morphologically indistinguishable from developmental outgrowth of wild-type AVM axons, as most axons reach the maximum length of the developmental neurons (Fig 3I). The finding that the number of morphologically wild-type axons increases with time in diapause, while the number of truncated axons (whose distal axon segments have already degenerated) decreases, suggests repair of these truncated axons is unlikely to be caused by the fusion of severed distal and proximal axons that has been observed in other models of axon repair [46– 48]. Together, our data indicate AVM axons in *mec-4d* dauer animals are capable of complete morphological regeneration and full restoration of mechanosensation.

The ALM and PLM mechanoreceptors expressing the *mec-4d* degenerin (Fig 2F), as well as the PVC interneuron expressing the *deg-1 degenerin* (Fig 2I), the ALM after axotomy (Fig 2H), and the ASJ sensory axons after injury [49] all regenerate in dauer animals, suggesting that diapause may promote regrowth of a broad number of neuronal types in response to various forms of injury. Beyond neuronal tissues, it will be important to study whether diapause can also promote the regeneration of other structures given that changes during dormancy are systemic. Interestingly, in the Colorado potato beetle the completely degenerated wing muscles fully regenerate during diapause [50].

### Diapause creates a propitious environment for neuronal regeneration

Factors that may play an important role in diapause-induced regeneration are low metabolic rate, increased function of pro-regenerative genes, and decreased function of genes that inhibit regeneration. A hallmark of dauers, non-ageing larvae, is the downregulation of the DAF-2 insulin receptor. Interestingly, axonal regeneration in aging motor neurons is inhibited by DAF-2, through regulation of the intrinsic neuronal activity of the DAF-16/FOXO transcription factor [29]. Congruently, our experiments show that *daf-2; mec-4d* mutant animals significantly regenerate the AVM neuron in non-dauer animals (Fig 6B). The protective and regenerative effects of DAF-2 downregulation in *mec-4d* dauers is likely mediated by DAF-16. We show that DAF-16 overexpression maintains neuronal integrity (Fig 6E), while *daf-16* mutation enhance degeneration of *mec-4d* neurons (Fig 6D).

Importantly, turning DAF-2 on in dauers decreases the number of animals with wild-type AVM and ALM axons (Fig 5C). This suggests that DAF-2 downregulation in diapause is upstream of several changes in protein activity and gene expression underlying axonal regeneration, likely triggered by the activation of DAF-16 among other changes. DAF-16 has a large number of evolutionarily conserved transcriptional targets, including genes involved in cellular stress-response, antimicrobial and metabolic genes [51– 53]. Importantly, expression of DLK-1, a key regulator of regeneration, is regulated by DAF-16 in neurons and downregulated by insulin/IGF1 signaling [29].

The extent of axon regeneration in dauers is larger than the extent of axon regeneration observed in *daf-2(ts)* mutants during normal development (Figs 2C and 6B). The increased regenerative ability could be a consequence of the incomplete downregulation of DAF-2 in the temperature sensitive mutant or due to the influence of other gene expression changes that occur independently of insulin pathway function.

### Maintenance of neuronal integrity during diapause

Once AVM axons have regenerated, the wild-type morphology is maintained until day 60 of diapause (Fig 2C). Likely, diapause creates a systemic and cellular environment that, in addition to enhancing axon regeneration, also protects neurons from degeneration even when the *mec-4d* prodegenerative stimulus is active. This protection is in part explained by the activation of DAF-16 and downstream transcriptional targets such as catalases and superoxide dismutase enzymes [54], which confer a high antioxidant capacity to dauer larva, and collectively inhibit neuronal degeneration [3]. Other relevant factors that contribute to the protection of *mec-4d* axons in diapause remain to be discovered. It will be important to study the electrophysiological properties of the MEC-4d channel in dauer quiescence to better understand the cause of the lack of degeneration. The ability of dauer animals to respond to mechanical stimulation suggests the channel may be functional (Fig 3D and 3E). Additionally, the amount of MEC-4 expression in diapause and in non-dauer larvae is very similar [3], suggesting that diapausing neurons have a strong ability to maintain cellular homeostasis.

### Neuronal somas suffice for diapause-induced regeneration

Individual analysis of *mec-4d* AVM regeneration showed that neurons with only a soma at dauer entry display the highest regenerative capacity (94 μm in average) compared to neurons with truncated axons (47 μm, Fig 3H). This d*e novo* growth of axons offers a new paradigm to study neuronal regeneration. Dauer somas and their extracellular matrix may resemble a stem cell like environment where the extension of an axon occurs anew. Interestingly, a number of anti-regenerative genes are downregulated in dauers. For example, *lin-12*/Notch, which limits neurite extension during development [55] and impairs regeneration [56], is down-regulated 1.9-fold in dauers (see S1 Table in [57]).

The smallest amount of growth was observed when axons are long at diapause entry (30 μm of average growth), especially in axons connected to the nerve ring. This is consistent with the finding of Wu et al. [12] where the synaptic branch inhibits regrowth of ALM and PLM touch receptor neurons. Animals whose neurons did not regenerate were those whose soma degenerated entirely before dauer entry. These animals did not display *de novo* AVM neurogenesis (S4 Table).

### Functional regeneration

The most favorable outcome of axonal repair is to regenerate circuits and to restore nervous system function. Few models of regeneration in invertebrates and mammals are able to attribute recovery of function to a specific regenerated axon. Examples in *C*. *elegans* are the recovery of movement observed after the injury induced in the D-type motor neurons [11, 56, 58], and the recovery of the posterior touch response after axonal fusion of injured fragments of PLM [31, 59].

In this work we used the term functional regeneration for those neurons that were capable of providing a response to anterior gentle touch after regrowth. The ALM, AVM, and PLM mechanosensory neurons sense touch to the body and provide input to the command neurons via both synaptic connections and gap junctions [60]. The anterior ALM and AVM touch cells are connected by a gap junction, which creates an anterior touch cell network. AVM neurons also function independently of the ALM neurons, presumably via the gap junctions with AVD and the synaptic connections with AVB [61]. The AVM neuron alone is capable of providing one or two responses out of ten to anterior touch [3, 60]. Combining the response to one anterior touch as a measure of AVM function with visualization of AVM morphology (MEC-17::GFP), we found dauer animals are capable of functional and morphological regeneration of the touch circuit (Fig 3E).

### DLK-1 dependent and independent growth

DLK-1 is widely required for regeneration of axons in worm, mice and fly models of axon damage [32–35]. Additionally, though seemingly contradictory, DLK-1 has been shown to have a role in the degeneration of axons in mice and flies [37, 38]. Here we show that after damage caused by hyperactive degenerins, DLK-1 is necessary for both degeneration when animals feed *ad libitum* and for complete morphological regeneration of the axons during diapause (Fig 8D). DLK-1 responds to axonal damage participating in at least two processes. The first includes the stabilization of the CEBP-1 BZip transcription factor and upregulation of genes required for axon regeneration such as poly-ADP ribose glycohydrolases [13, 36, 58, 62]. The second is a regulation of microtubule dynamics in *C*. *elegans* [13, 63, 64] that includes downregulation of the microtubule depolymerase KLP-7 and upregulation of the cytosolic carboxypeptidase CCPP-6, which promotes microtubule growth and axon regeneration [65]. Whether DLK-1 regulates protection and regeneration of *mec-4d* axons in diapause through similar mechanisms is an interesting avenue of future investigation.

We did observe weak DLK-1 independent regeneration at dauer entry and during the first 3 days in diapause. Upon individual examination, a 19.6% increase in growth at the third day was observed mostly when AVM neurons entered diapause with only a soma. This growth was always incomplete, and half of the time erratic (Fig 8A and S9 Table). This is consistent with the finding of DLK-1 independent outgrowth of the ASJ sensory neurons [49], and dendrite regeneration in *Drosophila* dendritic arborization neurons [66]. A possible explanation for the observed outgrowth, could be the action of the MAPKKK MLK-1 (*Jnk* pathway), which is essential for axonal regeneration [32, 67]. MEK-1, downstream of MLK-1 can activate PMK-3, and consequently generate a DLK-1-like activation [67], bypassing the need for DLK-1. These data suggest that even in the case that DLK-1 is inactive, there are other ways by which the *mec-4d* neurons can activate axonal outgrowth, but DLK-1 is necessary to achieve extended axon regeneration.

### A new model to study regeneration

In this work we use a degenerin model to study neuronal regeneration during diapause. Even though *mec-4d* is a chronic insult, we show that the degree of damage can be modulated by temperature.

This degenerin model has previously been demonstrated to be molecularly similar to mammalian Wallerian degeneration because it shares at least three molecular characteristics: it depends on intracellular Ca^2+^ increase, it is delayed by blocking the mitochondrial transition pore (mPTP) and reduced by overexpression of *nmat-2*, the *C*. *elegans* NMNAT-2 protein [3].

The relevance of this work is two-fold. First, we report that dauer quiescence is capable of functionally regenerating damaged axons. The changes that animals undergo are similar to other forms of suspended animation such as hibernation in mammals [68]. Diapause is capable of inducing full regeneration of neurons starting from a soma alone, and this regrowth is more extensive than from truncated axons. This implies that repairing a broken axon is more difficult for the cellular machinery then creating one anew. It will be interesting to explore what metabolic and molecular determinants of diapause underlie this powerful ability to regenerate neurons.

Second, diapause-triggered regeneration is genetically encoded, avoiding laborious interventions to damage axons. Furthermore, diapause induction can be performed by food depletion in order to obtain large numbers of injured animals. Finally, a behavioral test can easily be used to test the functionality of the neuron. Functional regeneration but not neuronal outgrowth in diapause depends on DLK-1, creating a broad platform for gene discovery both *dlk-1*-dependent and independent. This model also offers the possibility of electrophysiological examination of the MEC-4d channel *in vivo*, to understand its gating properties, ion selectivity under degenerative conditions, and mechanical stimulation. It will be important to investigate the metabolic changes that define a protective environment as well as the metabolic profile of neurons during diapause to better understand cell autonomous and systemic conditions that create an ideal environment for neuronal repair.

## Materials and methods

### *C*. *elegans* growth and maintenance

*C*. *elegans* wild type (N2) and mutants TU2773 [*uIs31(P*_*mec-17*_*mec-17*::*gfp);mec-4d(e1611)X*], TU253 [*mec-4*(*u253*)X], QW1314 [*dlk-1(km12); uIs31(P*_*mec-17*_*mec-17*::*gfp); mec-4d (e1611)X*], KU12 [*dlk-1(km12)*], QW1314 *[dlk-1(km12); uIs31(P*_*mec-17*_*mec-17*::*gfp)*], WCH34 [*daf-2(e1368ts)III; uIs31*(*P*_*mec-17*_*gfp*); *mec-4d(e1611)X]*, TU38 [(*deg-1(u38)*] and TU3755 [*uIs58* (*P*_*mec-4*_*mec-4*::*gfp*)], WCH36 ([*zIs356* (*daf-16p*::*daf-16a/b*::GFP; *rol-6(su1006)*]*; uIs31(P*_*mec-17*_*mec-17*::*gfp)*, *mec-4d (e1611)X)*, and WCH39 [*daf-16(m27)*, *uIs31(P*_*mec-17*_*mec-17*::*gfp)*, *mec-4d (e1611)X*] were maintained at 20°C in Nematode Growth Media (NGM) agar plates, inoculated with *E*. *coli* OP50 [69]. The expression pattern given by *P*_*mec-17*_*mec-17*::*gfp* is referred in the text as *mec-17*::*gfp* and that of *P*_*mec-4*_*mec-4*::*gfp*, *as mec-4*::*gfp*.

### Bacterial growth

*E*. *coli* OP50 was grown fresh from a -80°C glycerol stock, and streaked onto a Luria Bertani (LB) plate containing Streptomycin 25 mg/ml at 37°C. Next morning, a chunk of the lawn was allowed to grow for 6 hours in liquid LB with Streptomycin 25 mg/ml. 300 μl of the culture was seeded onto NGM plates and allowed to dry for 18–24 hours before use.

### Hypochlorite treatment

Large amounts of gravid adults were washed off plates with M9 buffer, collected in Eppendorf tubes and centrifuged for 2 minutes at 2500 rpm. Supernatant was discarded and the pellet resuspended in 1 ml of alkaline hypochlorite solution (20 ml of 1M NaOH, 30 ml NaClO and 50 ml H_2_O in 100 ml of solution). Tubes were kept at mild agitation and every 30 seconds an aliquot was removed for observation. Once only embryos remained (within 5 minutes from hypochlorite addition), tubes were centrifuged for 2 min at 2500 rpm and the supernatant discarded. The pellet was washed twice with M9 buffer.

### Criteria for neuronal integrity

#### AVM neuron

Morphological evaluation of dauers was modified from Calixto et al. [3]. Neurons with full-length axons as well as those with anterior processes that passed the point of bifurcation to the nerve ring were classified as AxW (see Fig 2A). Axons with a process connected to the nerve ring were classified as AxL, and those that did not reach the bifurcation to the nerve ring as AxT. Lack of axon was classified as AxØ and a soma only as AxØ-S. AVMs with only the ventral projection were called AxØ-S as well. *dlk-1; mec-4d* animals with morphological defects in the shape or direction of neurites were classified as the closest general category (for example AxT), followed by the letter ***e*** for error (AxT-**e**).

#### ALM and PLM neurons

Neurons with full-length axons were classified as AxW. AxT were ALM neurons with axons that did not reach the bifurcation to the nerve ring and PLMs were axons that did not reach mid body. Neurons without axons or somas were classified as AxØ.

### Synchronization of animals

Plates with large amounts of laid eggs were washed with M9 to eliminate all larvae and adults. Within the next two hours, newly hatched L1 animals were collected with a mouth pipette and transferred to the desired experimental plates.

### Time course of degeneration in non-dauer animals

Synchronized L1 larvae were placed in plates with food using a mouth pipette. The integrity of AVM axons at 12, 24, 48 and 72 hours post hatching was observed and scored. The same experiments at 25°C also included ALM and PLM neurons. For experiments at 25°C without temperature shifts, parentals of animals examined were kept at the same temperature starting as L4s. Animals were scored until 168 hours at intervals of 24 hours (Fig 1B). For each evaluation three plates with 30 worms were used.

### Microscopy

Dauer and non-dauer animals were placed on 2% agarose pads on glass slides, immobilized with 1 mM or 20 mM levamisole respectively. The preparation was covered with glass coverslips and subsequently observed under a 40x or 60x oil objective in an upright Nikon Eclipse Ni-U fluorescent microscope. For high resolution images in Figs 3I and 4G, we used a Leica TCS SP5X microscope.

### Photography

All images were taken with a Canon EOS Rebel T3i camera, attached to the Nikon Eclipse Ni-U microscope. Fluorescence photographs were taken with 1/10 sec exposure time, and ISO 3200. Nomarski (DIC) images were taken with 1/5 sec of exposure time and ISO 200.

### Killing of *E*. *coli*

NGM plates were seeded with 300 μl of an *E*. *coli* OP50 culture and allowed to dry for 18–24 hours. To kill bacteria, plate and lid were placed upside down on a UV-transilluminator (LabNet International, 100-240V, 50–60 Hz) for 5 minutes. To confirm that bacteria were dead, a portion of the lawn was streaked on an LB plate and allowed to grow overnight at 37°C.

### Dauer isolation

Dauers were isolated by 1% SDS treatment of animals in starved plates [5]. Plates were washed with 2 ml of 1% SDS, and transferred to an Eppendorf tube. The tube was agitated manually every 2 minutes for 15 minutes total. Finally, the tube was centrifuged for 2 min at 2500 rpm, the supernatant eliminated and the dauer pellet seeded on a plate without bacteria. To further isolate dauers from worm carcasses, live animals were allowed to crawl off the pellet for 30 to 60 minutes. The portion of the plate with the remaining pellet was excised from the plate with a scalpel and dauers collected either by pipette or a filtered pipette tip using sterile M9 with. This was performed at 20 and 25°C.

### Dauer synchronization

#### First day of dauer

A large number of embryos from hypochlorite treatment were placed on plates containing 300 μl of UV killed *E*. *coli* OP50. When the bacterial lawn was almost exhausted, plates were observed daily on a stereomicroscope Nikon SMZ-745 for the appearance of dauers. Once dauers were observed, animals on the plate were collected and the pellet treated with SDS 1% to isolate dauers on the earliest possible day (first day of dauer). Since normally there were only a few dauers the day of food exhaustion, dauers were collected from several plates to get as many synchronized first day dauers as possible. This was performed at 20 and 25°C.

#### Population-based dauer evaluation

Daily for one week: To monitor AVM neuron behavior every 24 hours, large population of dauers were synchronized in day 1, placing 30–40 dauers per well on a 24-well plate. At least 30 animals were used per triplicate per day. For daily morphological evaluation, animals were collected of three wells on an Eppendorf tube, and centrifuged for 2 minutes at 2500 rpm, carefully eliminating the supernatant. Animals were discarded after. The same procedure, performed at 20 and 25°C, was repeated 6 more times until the week was completed.

One-week and one-month old dauers: In these experiments the critical step was that each replica of 30 animals derived from the same plate. To this end, the collection was delayed to the second day of dauers, when the number of animals was large enough. Once the required number of animals was obtained, SDS treatment was performed and the pellet placed in a plate without bacteria. Dauer animals that moved out of the pellet were collected with a mouth pipette placing 30–50 individuals per well on a 24 well plate to be evaluated after a week or a month.

More than one-month dauers: The starting population used for dauers older than one month derived from embryos collected after hypochlorite treatment. Once the first day of dauer was established after food exhaustion, plates were sealed and kept unopened for 60 or 90 days. After the desired time, plates were treated with SDS 1% to collect and analyze the morphology of the AVM neuron of dauers. Plates were strictly controlled to be clean of *E*. *coli* OP50 food or contamination of any kind. Importantly, another requisite was that the first day of dauer, plates did not contained animals younger than L3 to avoid their entrance into diapause at later times.

### Individual and longitudinal analysis of dauers

To directly observe the regenerative effect of diapause entry, individual dauers were followed in a time dependent manner. Two factors were critical in this protocol: 1) avoiding contamination in order to keep worms in dauer, and 2) obtaining animals in the first day of dauer. To avoid contamination, a) collected dauers were descendant of parental embryos from hypochlorite treatment, fed on previously UV killed *E*. *coli* OP50 (see above); b) SDS 1% was supplemented with carbenicillin 25 mg/ml (Santa Cruz Biotechnology) and 250 mg/ml of Amphotericin B (fungizone, Gibco/Thermofisher).

One-day dauers were obtained by SDS 1% treatment. The pellet was washed twice with H_2_O containing the same antibiotics mentioned before, under agitation for another 10 minutes (5 minutes with each wash). The total pellet was placed on the center of an empty NGM plate, and dauers were allowed to crawl outside the pellet for collection. The fraction of the plate that contained the carcasses and the total pellet was cut out using a scalpel or stainless laboratory spatula. Dauers that were on the remaining agar, were collected with H_2_O, centrifuged and seeded on a new NGM plate without food. Animals were individually collected with a mouth pipette and placed on an agarose pad with 20μM levamisole for observation. This protocol was performed at 20 and 25°C.

### Criteria for selection of individual dauers

To be considered for individual evaluation, dauers had to have a degree of axonal truncation on the first day, including incomplete AxW (second image in Fig 2A) and animals with only a soma AxØ-S or no visible neuron (AxØ). Each animal was mounted on 2% agarose pad using a mouth pipette and levamisole 20 μM. If animals fulfilled the above morphological criteria, they were photographed under fluorescence and Nomarski (DIC) in the same position, in order to collect information from the first day and compare it to the following days. After image collection, animals were taken from the agarose pad. Using a scalpel, the coverslip was separated from the agarose bed, avoiding breaking or losing the worm as a consequence of detaching the glass. After this, using a mouth pipette and a mix of autoclaved distilled H_2_O supplemented with Carbenicillin (25 mg/ml) and Amphotericin B (250 mg/ml), animals were collected from the pad and placed on individual wells in a 24-well plate. Each well contained 200 μl of H_2_O plus Carbenicillin (25 mg/ml) and Amphotericin B (250 mg/ml) and 30 wild type (N2) dauers (see explanation below). These dauers were obtained using plates of N2, which were starved for at least two weeks, being the main criterion the presence of large quantity of dauers.

24-well plates were sealed with parafilm (Bemis, Parafilm M) and incubated at 20 and 25°C without agitation until the second evaluation 2 days later (day 3 of dauers). On day 3, the content of the well was collected with filtered tips on an Eppendorf tube. Animals that remained on the edges of the well were collected with a mouth pipette. Tubes were centrifuged for 4 min at 2500 rpm. Most supernatant was carefully discarded and the remaining pellet was placed on an agarose pad. With a mouth pipette any drop left on the Eppendorf tube was extracted. Finally, 20 μM levamisole were added to paralyze all dauers. To avoid dispersion caused by covering the preparation, the coverslip was placed once the liquid was absorbed. *gfp* expressing animals were located among all dauers and registered as described before.

**Explanation for mixing each *mec-4d* dauer with wild type dauers:** Maintaining individual dauers was challenging. When growing them on plates after 24 hours, they either escaped or were lost under the agar. In liquid, individual dauers tended to enlarge the pharynx and started pumping, and displayed morphological changes proper of dauer recovery, even though they were unable to resume development for the lack of food. We reasoned that mixing the *mec-4d* dauer with a population of other dauers would prevent them from dauer recovery (for the hormonal effect). To distinguish them, N2 dauers without any visible marker were added to each *mec-4d* animal express *gfp* in the AVM cell.

### Dauer observation at 25°C

*mec-4d* eggs, obtained from hypochlorite treatment, were grown at 20°C until the L4 larval stage. Plates were then moved to 25°C, where the F1 hatched and develop. Once bacteria were consumed, the plate was observed for the appearance of first day dauers. If one dauer was found, plates were treated with SDS 1% as explained above.

### Dauer temperature shifts

To evaluate the effects of DAF-2 downregulation on dauer animals, we used the strains WCH34 (*daf-2ts(e1368); uIs31 (P*_*mec-17*_*mec -17*::*gfp); mec-4d(e1611)* and TU2773 [*uIs31(P*_*mec-17*_*mec-17*::*gfp);mec-4d(e1611)X*] as a control. Embryos from both strains obtained from hypochlorite treatment, were placed on plates at 25°C until diapause entry by starvation. SDS 1% treatment and dauer scoring was performed as described for *one-week dauers* above. 30–50 dauers were used to record the morphology in the first day as dauer at 25°C. Then, animals were divided into three plates and shifted to 15°C and 20°C. Morphology of AVM and ALM was scored 2, 4 and 6 days after the temperature shift.

### Dauer recovery

One-week old dauers that derived from the same plate were obtained by SDS 1% treatment. The first evaluation was done with 30 dauers directly from SDS 1% treatment to establish the hour zero of dauer recovery. 30 to 50 dauers were placed on 3 plates with *E*. *coli* OP50 and examined every 24 hours until 96 hours post-dauer.

### Determination of lifespan in diapause

To measure the lifespan of wild type and *mec-4d* animals in diapause, large amounts of dauers were isolated using the same criteria for the synchronization of dauer explained above (*More than one-month dauers*). Minimally 30 dauers we placed in each well of a 24-well plate, filled with distilled water, antibiotics and antifungal as mention above. The media of each well was changed with new liquid every two weeks. The quantification of dauers was done using three wells (with at least 30 dauers each) per week per strain for a total of 16 weeks. Each well was labeled with the number corresponding to the week its examination was scheduled. To evaluate the number of live dauers, animals were collected from each well and placed on a 60mm NGM plate with *E*. *coli* OP50 food. Total dauers were immediately counted to record the initial number of individuals. 24 hours later, the number of live non-dauer animals was counted again. For each time point the percent of live animals was calculated using the initial dauer count. Each point in the graph is the average of three replicas.

### Individual and longitudinal analysis of developing animals

To directly observe the regeneration of the embryonic neurons in developing animals at 25°C, individual animals were followed during the initial 24 to 48 hours of development. Animals were selected based on the criteria of having truncated axons, according to the morphological categories described above for ALM and PLM neurons. Newly hatched L1s were collected with a mouth pipette and mounted on agarose pads immobilized with 1 μM levamisole. Worms were observed and photographed in the microscope under 60x objective to keep a registry of the morphology of the ALM/PLM neurons at every time point. Then, animals were rapidly passed to a new plate with *E*. *coli* OP50 food. 24 and 48 hours later the same protocols were repeated. Animals were maintained at 25° at all times.

### Touch test

To evaluate the functionality of the AVM mechanoreceptor neuron, the ability of dauer animals to respond to gentle touch was tested [26]. Animals were touched at 20 and 25°C.

#### Touch test of dauers during regeneration

Synchronized dauers for daily analysis during one week, were maintained in groups of 30–50 on wells of a 24-well plate. Each day, animals were taken from a well, collected in an Eppendorf tube, centrifuged at 2500 rpm and placed back on an empty NGM plate. Once the liquid evaporated, animals were allowed to crawl out of the pellet for about an hour before the touch test. *mec-4d* dauers were touched with an eyebrow one time in the head, gently stroking where the pharyngeal bulb lies [3].

#### Touch test of non-dauers

L4 and adult animals were gently touched with an eyebrow 10 times in a head to tail fashion [26].

### Correlation between morphology and function

To correlate AVM morphology with the ability of animals to respond to the anterior touch, touch tests were first performed to dauer animals synchronized as explained above and immediately mounted on agarose pads for observation in the microscope and scoring of the AVM morphology. This protocol was performed at 20 and 25°C.

### Measurements of dauers and image analysis

#### AVM neuron size

To quantify the growth of the AVM, ALM and PLM neurons during diapause, photographs taken under fluorescence and Nomarski were analyzed using ImageJ. The following measures were taken with the Straight-Line tool: Length of the anterior process of the AVM the first and third day of dauer, ii) Distance from the distal end of the anterior AVM process to the nose. Neubauer chamber was used to calibrate the equivalency on microns of the images in the program. All photos were formatted to a resolution of 5184 x 3456 pixels. To accurately classify and compare the different morphological features of neurons, the following features were established: **Max AxW** is the maximum length of the AVM axon. To estimate the expected maximum size of a given axon, we measured the average distance between the tip of the nose and the tip of the AVM axon of 30 wild type dauer animals expressing *gfp* in the touch neurons [*uIs31* (*P*_*mec-17*_*mec-17*::*gfp*)]. This distance was 5 μm. Finally, **Max AxW** for each animal evaluated was obtained by measuring the distance from the distal part of the remaining axon, to the tip minus 5 μm. **Size respect to Max** was calculated considering the initial length of the axons at day one as a percentage of the the Max AxW. **Growth** was calculated subtracting the length at day 1 from the final length of the axon at day 3. **growth (%)** was generated comparing the growth in relation to the Max AxW.

#### Pharynx

To accurately determine whether animals remained as dauers for the duration of the protocol, the width of the terminal bulb (diameter passing through the grinder, transversal to the body) was measured. The criterion is that the diameter does not change more than 5 microns between the first and third day.

### MEC-4 channel quantification

To quantify the number of MEC-4 channels in the TRN axons, the strain TU3755 containing the *uIs58* (*P*_*mec-4*_*mec-4*::*gfp*) transgene was used. MEC-4 channels appear as puncta along the axon and can easily be counted [70] Specifically, the PLM neuron was used to measure the number of puncta in synchronized L2, day 1 and day 7 dauers and 24 hours after dauer recovery, at 20 and 25°C. Once the animals were in the required state, each animal was mounted on an agarose pad and immobilized using 1μM levamisole. Then, animals were photographed focusing on the PLM neuron taking different focal planes of the axon, to obtain the best representation of the MEC-4 channels. From each state, 20 animals were scored. Pictures were analyzed with ImageJ, using the Neubauer chamber to calibrate the equivalency on microns of the images in the program. All photos were formatted to a resolution of 5184 x 3456 pixels.

To normalize the measurements and consider the same size in all animals, PLM soma was taken as initial point and from this 120 μm was measured. The quantity of channel found in this axonal size was quantified and taken as value for each animal. This procedure was repeated for each animal in all state evaluated. To synchronized dauer we performed the same protocol mention above.

### Temperature shifts

To evaluate the effect of DAF-2 hypofunction on the regeneration of the AVM neuron, we used the WCH34 strain [*daf-2ts(e1368); uIs31 (P*_*mec-17*_*mec -17*::*gfp); mec-4d(e1611)*]. L1 were synchronized by mouth pipetting from plates at 20°C (0–2 hours post hatching) and transferred to 15 different new plates at 15°C or 25°C (30 plates total, 30 L1 per plate minimally) and allowed to grow for 24 hours. Animals from 3 plates at each temperature (shifted to 15°C or 25°C) were used for morphological assessment at 48 and 72 hours post hatching which are 24 and 48 hours after the shift respectively.

### Morphological and functional evaluation of DAF-16 overexpression on AVM

To evaluate the effect of DAF-16 overexpression in the degeneration of the AVM mechanoreceptor, a *mec-4d* strain expressing the *zIs356* transgene [*daf-16p*::DAF-16A/B::*GFP; rol-6(su1006)*] was created (WCH36) Due to the high intensity and diffuse expression pattern of the *zIs356* transgene, usual observation of AVM axons as integrity assessment was not possible. To circumvent this, we used two different approaches. Functionality of the AVM neuron was assessed by a behavioral touch test [26] in both TU2773 and WCH36 strains. Secondly, animals were scored in an Epi-Fluorescence Stereoscope (AZ-100, NIKON) for the presence of neuronal somas as a readout of neuronal protection.

### Axotomy experiments

Axotomy experiments were carried out as previously described [71]. Post axotomy images were acquired with an Olympus DSU mounted on an Olympus BX61 microscope, Andor Neo sCMOS camera, and Lumen light source. Animals were scored 18–24 hours after axotomy.

### RNA extraction and reverse transcription

Total RNA was isolated using TRIzol (Invitrogen) from N2 and *mec-4d* animals, in L2 and dauer stage. 100 μM of RNA were used for each reverse transcription reaction to using Superscript IV reverse transcriptase (Thermofisher Scientific).

### Quantification of *dlk-1* isoforms

To determine the quantity of both isoforms of *dlk-1* transcripts, a semi-quantitative PCR was performed using *ama-1* as reference of constitutive expression. Cycle number was standardized to remain in the exponential phase of amplification. Amplification was performed using KAPA Hifi HotStart ready mix PCR (KAPA Biosystems). 100ng of DNA were used for each sample as template for all conditions. Primers used were for *dlk-1*a, forward 5’^,^ CTTGGTCACACCAACATCAG 3’ and reverse 5’^,^ GTGTCACAAGCTCCGACC 3’; for *ama-1*, forward 5^’^ GTGGATGCGGTCATCAACCATC 3^’^ and reverse 5^’^ TTCTTTTCCTTTCAGTCGCTGCTTG 3^’^. 25 μL and 10μL of reactions were used to run on a 3% agarose gel for *dlk-1* and *ama-1* samples respectively. Quantification of band intensity was done using ImageJ, taking *ama-1* as control of a constitutively expressed gene.

### Biological and technical replicates

Each experiment was performed in three technical triplicates and at least three biological replicates. Biological replicates were defined as experiments made in different days, containing triplicates of each condition, and a technical replicate as a triplicate of the same condition on the same day. The average of the three reads of each triplicate was considered as one count of a minimum of three for each point of a curve. In other words, the data is collected and processed as a single technical replicate (the average of three counts of the same plate), and its mean is used as a single biological replicate. Each figure contains at least three experiments (biological replicates). Exceptions of that number per figure are: (1F) 653, (2B) 208, (2C) 918, (2G) 184, (3F) 160, (3G) 41, (4A) 240, (4F) 60, (6E) 120, (7D) 205.

### Criteria for data exclusion

Entire experiments were excluded when there was contamination with unwanted bacteria or fungi.

### Statistical evaluation

Statistical evaluation was performed using one or two way-ANOVA, with *post-hoc* tests, and Chi square test when indicated. Results of all tests are detailed in Supporting Information (S2 File).

## Supporting information

S1 Fig Time course of morphology of *mec-4d* PVM at 20°C during development. (TIF)

S2 Fig Survival of wild type and *mec-4d* dauers during 16 weeks in diapause. P values ****< 0.0001, ***< 0.001, **<0.005, * <0.05. Error bars indicate the SEM in at least three biological replicas done in triplicates which had 30 or more animals each.
(TIF)

S3 Fig High resolution confocal images depicting representative PLM neurons expressing *mec-4d*::*gfp* at different stages, at 20 and 25°C. Scale bars represent 20 μm.
(TIF)

S4 Fig Comparison of measures of pharynx between dauers of one day, one week, and one month with developing animals. (TIF)

S5 Fig Time course of morphology of AVM in *mec-4d* during development at 25°C. (TIF)

S1 Table Measurements of individual *mec-4d* AVM neurons on the first and third day of diapause. Size of axons is reported in μm. **Max AxW** is the length from the AVM soma until the tip of the nose of each individual minus 5 μm (in average wild type AVM neurons stop 5 μm from the tip of the nose, N = 30); **Size respect to Max** is the % of the Max AxW at the first day; **Growth** is the size of the first day subtracted from the size on the third day. **growth (%)** is the % growth of Max AxW. Red font indicates functional regeneration.
(XLSX)

S2 Table Pharynx size of *mec-4d* animals that regenerated AVM neuron. Width of the pharyngeal bulb measured on days 1 and 3 of diapause of *mec-4d* animals showing regeneration of the AVM axon. AVM data is reported on Fig 3G.
(XLSX)

S3 Table AVM size of *mec-4d* animals that did not regenerate. Measurements of individual *mec-4d* AVM neurons on the first and third day of diapause. Size of axons is reported in μm. **Max AxW** is the length from the AVM soma until the tip of the nose of each individual minus 2μm; **Size respect to Max** is the % of the Max AxW at the first day; **Growth** is the size of the first day subtracted from the size on the third day. **growth (%)** is the % growth of Max AxW.
(XLSX)

S4 Table Pharynx size of *mec-4d* animals that did not regenerate the AVM neuron. Width of the pharyngeal bulb measured on days 1 and 3 of diapause of *mec-4d* dauer animals. AVM data is reported on S2 Table.
(XLSX)

S5 Table AVM morphological assessment of *mec-4d* animals that started diapause without AVM soma. Neuronal categories on days 1 and 3 of diapause of selected dauers whose AVM had AxØ morphology on day 1.
(XLSX)

S6 Table Pharynx size of *mec-4d* animals that started diapause without AVM soma. Width of the pharyngeal bulb measured on days 1 and 3 of diapause of *mec-4d* dauer animals illustrated on S4 Table.
(XLSX)

S7 Table Embryonic cells in dauer at 25°C. Measurements of individual *mec-4d* ALM and PLM neurons on the first and third day of diapause. F, Number of animals counted on each category with a summary of their growth.
(XLSX)

S8 Table Embryonic cells in development at 25°C. Measurements of individual *mec-4d* ALM and PLM neurons at hatching, 24 and 48 hours after hatching. F, Number of animals counted on each category with a summary of their growth.
(XLSX)

S9 Table Measurements of individual *dlk-1; mec-4d* AVM neurons on the first and third day of diapause. Number of animals counted on each category with a summary of their growth.
(XLSX)

S10 Table Pharynx size of *dlk-1; mec-4d* animals. Width of the pharyngeal bulb measured on days 1 and 3 of diapause of animals illustrated on Fig 8D.
(XLSX)

S11 Table Touch responses not reported in Fig 2B. (XLSX)

S1 File Raw data with replicas of all the experiments shown in the manuscript. (XLSX)

S2 File Statistical analysis of the experiments contained in the manuscript. (XLSX)
