# Supplementary figures and images for: Diapause induces functional axonal regeneration after necrotic insult in C. elegans
Source: PLoS Genet. 2019 Jan 14;15(1):e1007863. doi: 10.1371/journal.pgen.1007863 (PMC6347329; doi:10.1371/journal.pgen.1007863)

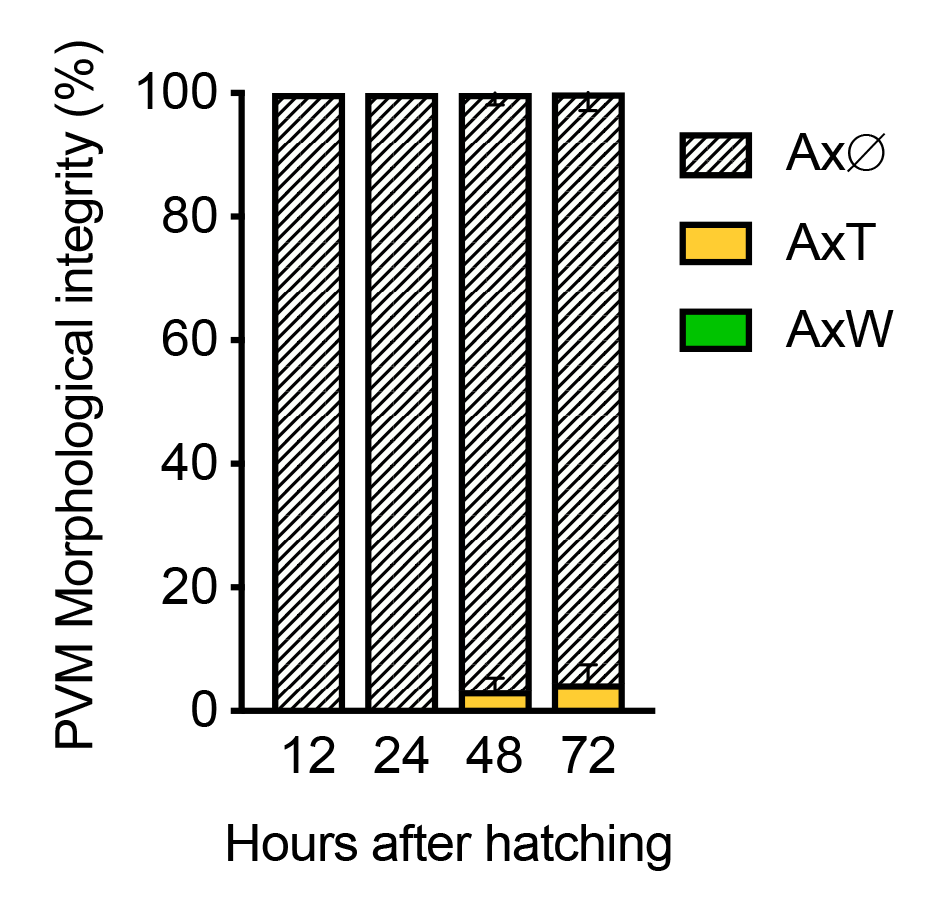

Supplement: S1 Fig — (TIF) [file pgen.1007863.s001.tif]

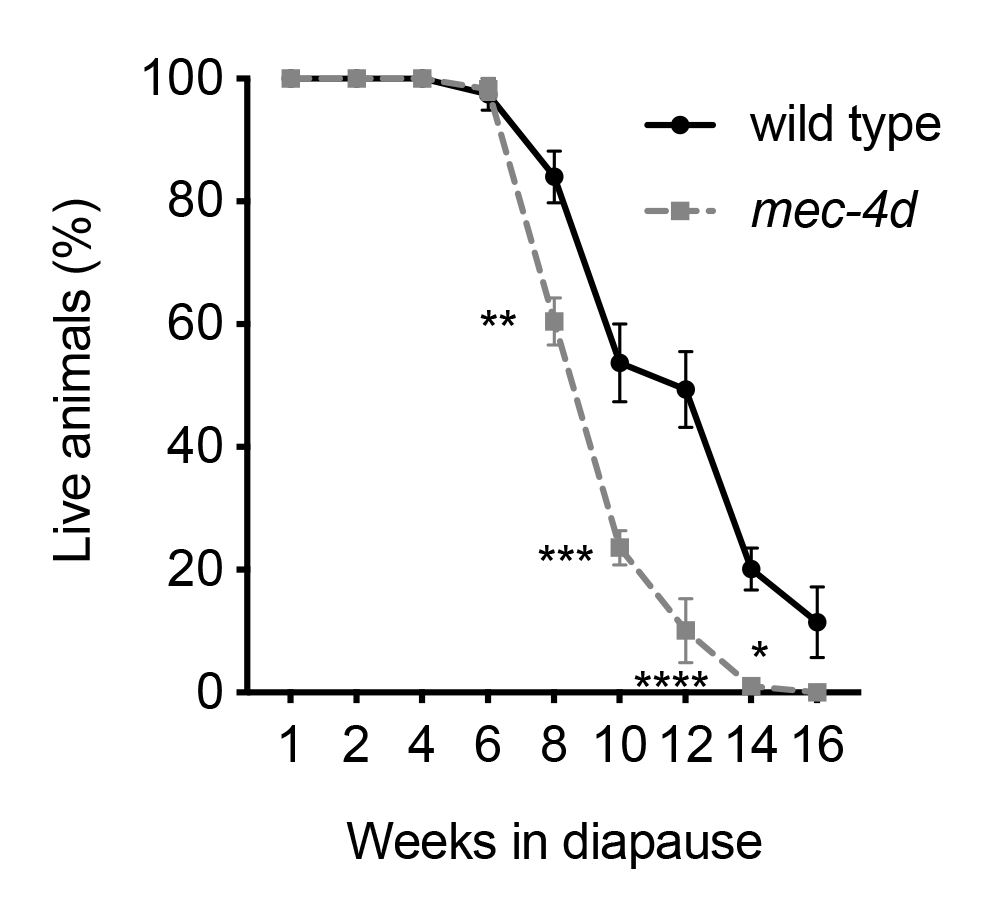

Supplement: S2 Fig — P values ****< 0.0001, ***< 0.001, **<0.005, * <0.05. Error bars indicate the SEM in at least three biological replicas done in triplicates which had 30 or more animals each. (TIF) [file pgen.1007863.s002.tif]

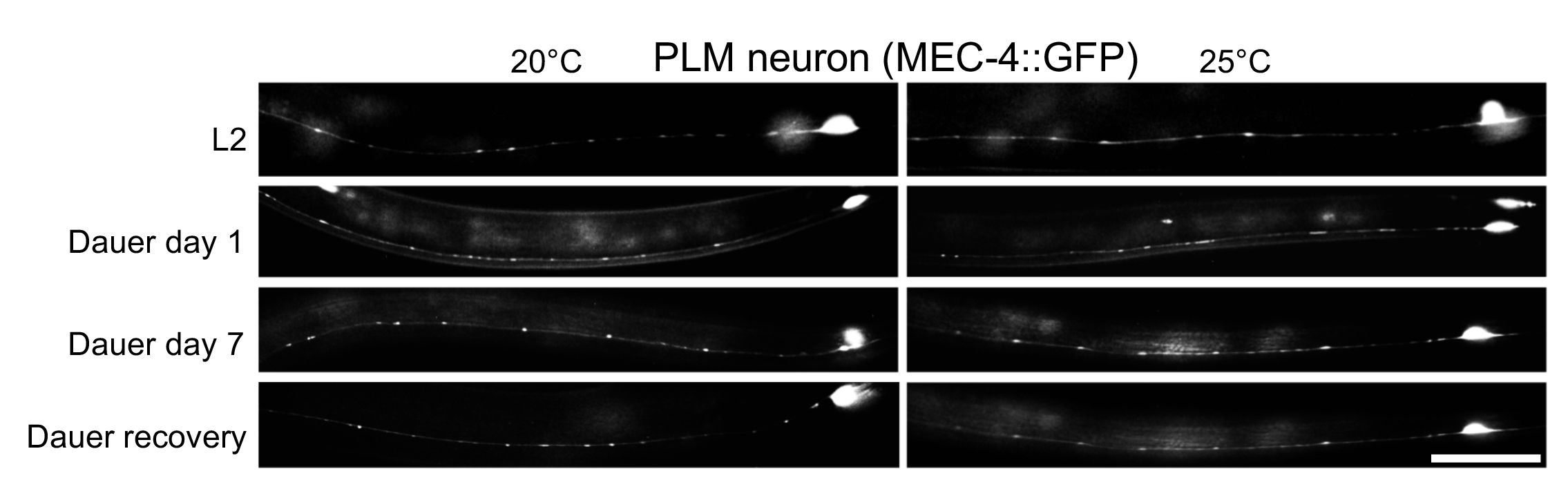

Supplement: S3 Fig — Scale bars represent 20 μm. (TIF) [file pgen.1007863.s003.tif]

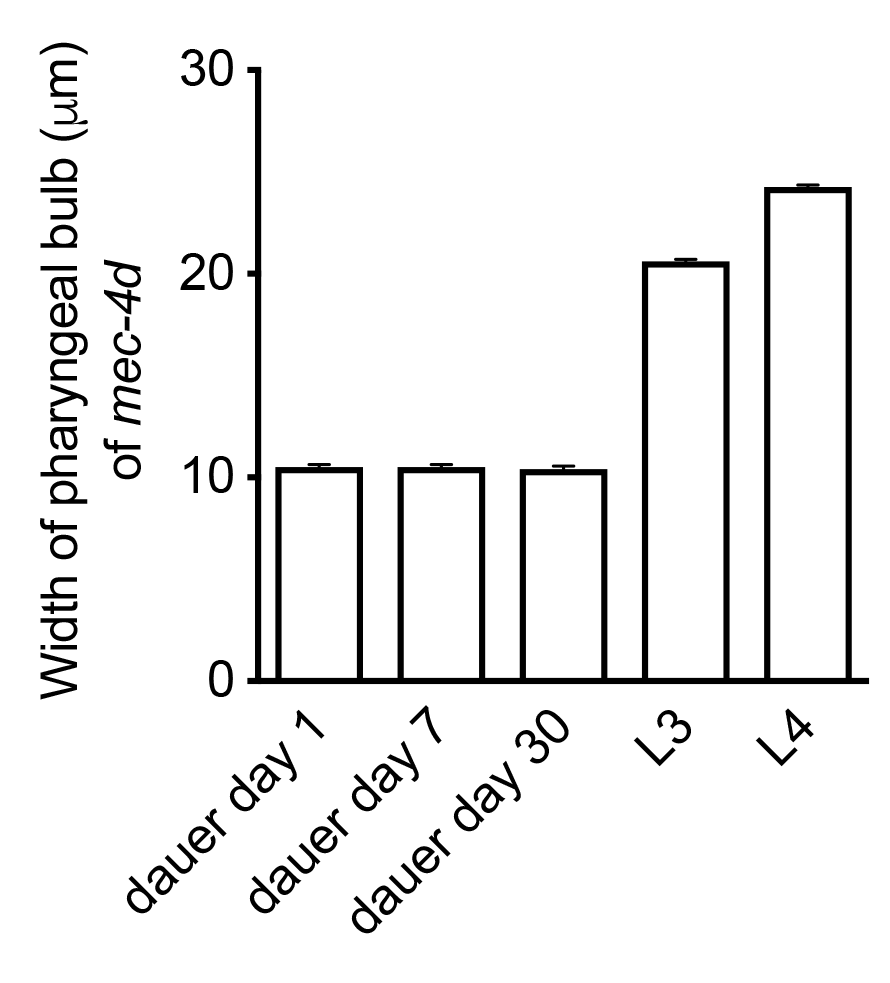

Supplement: S4 Fig — (TIF) [file pgen.1007863.s004.tif]

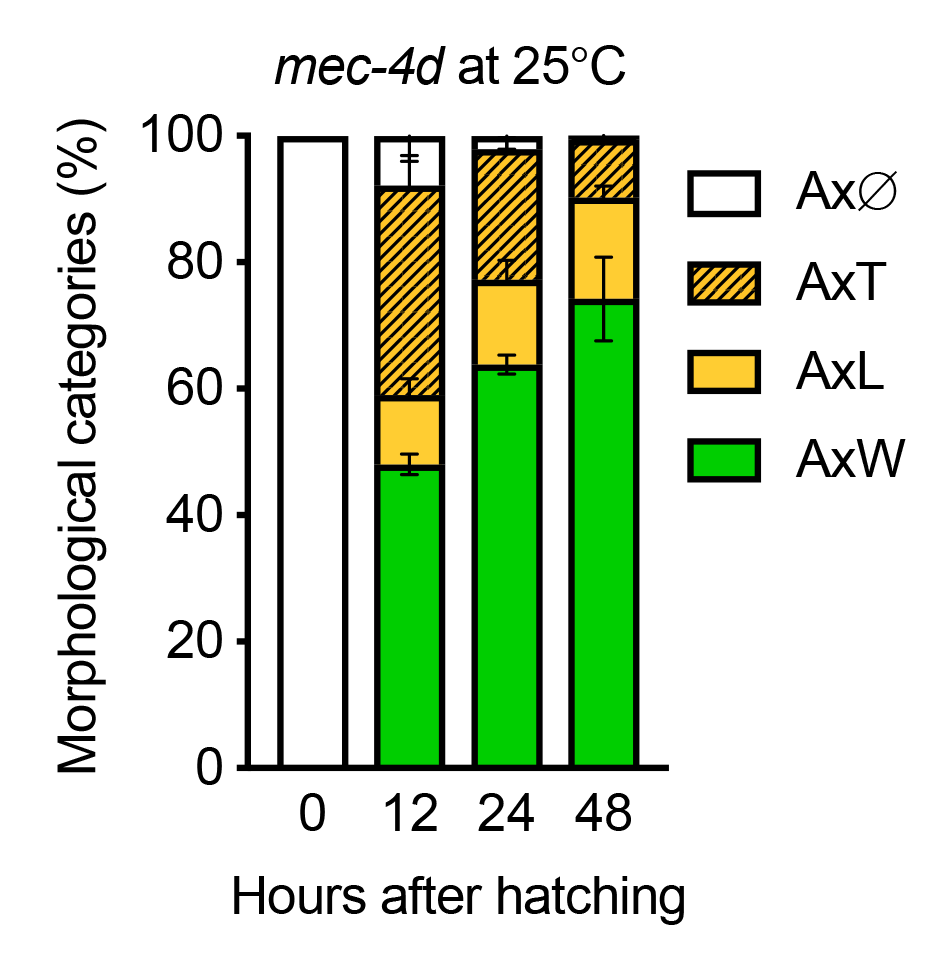

Supplement: S5 Fig — (TIF) [file pgen.1007863.s005.tif]
